# Elucidating Solvation Structures for Rational Design of Multivalent Electrolytes—A Review

**DOI:** 10.1007/s41061-018-0195-2

**Published:** 2018-04-26

**Authors:** Nav Nidhi Rajput, Trevor J. Seguin, Brandon M. Wood, Xiaohui Qu, Kristin A. Persson

**Affiliations:** 1 Lawrence Berkeley National Laboratory, Berkeley, CA 94720 USA; 2 Department of Materials Science and Engineering, University of California, Berkeley, CA 94720-1760 USA; 3 Graduate Group in Applied Science and Technology, University of California, Berkeley, CA 94720-1760 USA; 4 Joint Center for Energy Storage Research (JCESR), Chicago, USA

**Keywords:** Multivalent electrolytes, Solvation structure, Renewable energy

## Abstract

Fundamental molecular-level understanding of functional properties of liquid solutions provides an important basis for designing optimized electrolytes for numerous applications. In particular, exhaustive knowledge of solvation structure, stability, and transport properties is critical for developing stable electrolytes for fast-charging and high-energy-density next-generation energy storage systems. Accordingly, there is growing interest in the rational design of electrolytes for beyond lithium-ion systems by tuning the molecular-level interactions of solvate species present in the electrolytes. Here we present a review of the solvation structure of multivalent electrolytes and its impact on the electrochemical performance of these batteries. A direct correlation between solvate species present in the solution and macroscopic properties of electrolytes is sparse for multivalent electrolytes and contradictory results have been reported in the literature. This review aims to illustrate the current understanding, compare results, and highlight future needs and directions to enable the deep understanding needed for the rational design of improved multivalent electrolytes.

## Introduction

A key challenge of modern society is the sustainability of energy supply with increasing demand and depleting fossil resources. We are on the verge of a power revolution, where we desire enhanced energy storage devices with higher energy density, faster charge/discharge capability, and longer life compared to existing technologies. Access to low-cost and environmentally benign energy storage devices will not only transform the world’s energy economy but also build a foundation for a carbon-free society. Li-ion batteries are considered a linchpin technology of energy storage because of their ubiquitous use in electric vehicles and electronic devices. However, current demands are pushing Li-ion technology to the limit of its capacity, which is raising serious safety concerns [1]. Even with the enhancement in energy density by 5% per year and a reduction of the cost by 8% per year, the Li-ion chemistry is perceived as incapable of meeting the high energy density, long cycle life, and low-cost requirements for future electric vehicles and electronic devices due to inherent materials limitations [2]. Hence, exploration of the vast space beyond Li-ion batteries to identify potentially safer, cheaper, and environmentally benign battery technologies is warranted.

In this context, alternative technologies such as metal–air, redox flow, and multivalent batteries are investigated. Among several proposed post-Li-ion technologies, multivalent ion batteries have spurred renewed popularity in the last two decades owing to their theoretically high volumetric capacity, improved safety, and eco-friendliness as compared to state-of-art Li-ion batteries. As multivalent cations (Mg^2+^, Ca^2+^, Zn^2+^, Al^3+^, Y^3+^,…) proffer more than one electron per redox center, they are capable of delivering significantly higher energy density as compared to monovalent Li-ion and Na-ion batteries. Even though the concept of both divalent and trivalent batteries dates back to 1970s, rechargeable trivalent batteries remain in the realm of scientific curiosities [3, 4] and greater success of divalent chemistries abated the momentum of global research efforts on trivalent batteries [5–8]. A successful secondary multivalent battery requires a metal or metal alloy negative electrode, a high-potential cathode material enabling reversible intercalation, and a non-flammable electrolyte that can provide efficient transport of ions between anode and cathode while supporting the formation of functional solid–electrolyte interphase. A practical electrolyte enables both a stable solid-electrolyte interphase (SEI), as well as facile ionic transfer through the interface. In many conventional electrolytes, divalent ion metals react with the electrolyte to form truly passivating surface films, which inhibit ionic transfer. For example, most magnesium analogues of lithium salts and typical solvents used in Li-ion batteries undergo decomposition at the Mg metal surface, resulting in a passivation layer that is both electronically as well as ionically insulating, hindering the cell chemistry [5, 9]. This is in contrast to commercial Li-ion technology, where the graphite SEI, once formed, prohibits further reactions and supports Li-ion diffusion.

However, the limitations and design metric of different multivalent ions are quite different from each other. For example, Zn exhibits a less negative electrochemical potential as compared to the standard hydrogen electrode (− 0.8 V) than Ca (− 2.9 V) and Mg (− 2.4 V), however the volumetric capacity (5851 mA h/ml) of the Zn metal anode is larger than that of the more popularly studied Mg (3833 mA h/ml). Additionally, Zn metal is prone to dendrite formation upon cycling, limiting the lifetime and safety of a Zn-ion battery. This was reported early in the reversible plating/stripping of zinc metal with aqueous electrolytes in which limited cycle life was observed [10]. However, more recent reports have shown markedly improved cycling performance, poising aqueous Zn-ion batteries as promising candidates for grid applications due to lifetime, cost, and durability considerations [11]. Good compatibility of Zn, particularly with conventional non-aqueous electrolytes, has also been observed [12]. Mg metal is less prone to dendrite formation, which makes it a potentially safer candidate system as compared to Li and Zn [13]. One of the main advantages in multivalent systems originates from volumetric energy gains on both electrodes, which directly impacts the resulting size of the battery and hence cost/kWh [14]. However, reversible multivalent chemistry is fraught with challenges; the major obstacles in the commercialization of Ca-ion and Mg-ion battery are the lack of suitable electrode materials in which ions can be inserted and extracted reversibly and supporting electrolytes that demonstrate the optimal conductivity, electrochemical stability window, and chemical compatibility with both electrode materials. While multivalent cations generally exhibit sluggish solid-state diffusion in most close-packed structures, recent investigations point to host structures with non-preferred coordination landscapes to achieve optimal mobility [15, 16]. Furthermore, while Zn exhibits good cyclability in several electrolytes [12, 17, 18], and a few electrolytes show reversible plating and stripping of Mg metal [19–22], Ca has been proclaimed impossible for rechargeable energy storage applications [23]. However, recently, electrolyte formulations of Ca(ClO_4_)_2_ and Ca(BF_4_)_2_ in ethylene carbonate and propylene carbonate were demonstrated on Ca metal, albeit exhibiting approximately a 2-V overpotential at elevated temperatures (50–100 °C) [24].

Recently, the solvation structure and local dynamics of the electrolyte, as a function of the liquid components and concentrations thereof, has garnered increased attention in order to elucidate critically important phenomena for battery performance. For example, equilibrium ion association constants and diffusion coefficients impact ionic conductivity [25], as well as the formation and stability of the electrode–electrolyte interface [26], which in turn influences electrode stability and kinetics. In spite of the effort devoted to developing future electrolytes for multivalent batteries, there are still many unanswered questions regarding the intricate relationship between the electrolyte composition, structure, and dynamics, and the nature of the passivation layer formed at the metal interface. A desirable, rational designer approach to functional electrolytes requires a fundamental understanding of the local inter-molecular interactions, e.g., the solvation structure of the liquid solution and its impact on properties such as conductivity, viscosity, stability of the solution species, and resulting SEI. The term “outer sphere ion pair or ion pair” in liquid solutions is described for the oppositely charged species bonded together by simple electrostatic interactions, while species bound through short-range or covalent interactions in which a ligand temporarily donate a pair of electrons to fill an unoccupied orbital in the other atom (of neutral solvent molecule or negative ions) are described as “inner-sphere ion pair or complex” [27]. However, most methods of determining the association fail to distinguish between ion pair and complex formation. Also, since the solvated species are present in the solution through a series of equilibria, it is possible to observe simultaneous existence of different solvate species and complexes [27]. The outer sphere ion pairs are generally classified into four categories: (1) free ions, (2) solvent separated ion pairs (SSIPs), (3) contact ion pairs (CIPs), and (4) aggregates (AGGs). When both ions exhibit complete first solvation shells and do not share solvents, they are defined as free ions, however, when they share one or more solvent molecules, the solvate species is termed as SSIPs. When one to two and more than two counter-charged ionic species are present in the first solvation sphere of an ionic species, the ion pair is defined as CIP and AGG, respectively [28–30]. The true nature of the bonding is generally not known and some covalent character is expected to be present when ions are in close contact with each other. Both coulombic as well as covalent interaction energies contribute to the enthalpy term in free energy and the magnitude of enthalpy determines the extent of association between ions/molecules. Hence, the denomination of CIP and complex can sometimes be used interchangeably for the same species in the solution for e.g., MgCl^+^; similarly, higher AGGs are also termed as oligomers. Lastly, an ion pair complex is defined for a cationic and anionic complex that form an ion pair. In particular, when transition metal ions form a CIP or AGG with electron pair donors, they can form either cationic, anionic, or neutral complex. It is worth noting that when a monovalent salt (*Z* = 1) forms an ion pair, the net charge is zero with the formation of a CIP (e.g., [Li^+^-TFSI^−^]), similarly when a multivalent salt (*Z* > 1) forms an CIP the net charge is still zero (e.g., [Mg^2+^–SO_4_^2−^]), however when a multivalent cation (*Z* > 1) forms an ion pair with a monovalent anion (*Z*′ = 1) the net charge is (*Z*–*Z*′) q (e.g., [Mg^2+^-TFSI^−^]^−^) [31].

It is well known that the structures of the active species in solution can significantly affect the kinetics of metal deposition and dynamics of charge-carrying species. In conventional electrolytes, the formation of ion pairs and AGGs are generally assumed to negatively affect the mobility of ions and the transference number, which in turn decreases the conductivity [32]. On the other hand, there is speculation that formation of CIPs in Li salts with low dielectric solvents can increase the overall dielectric constant of the electrolyte, improve solvation of free species at higher concentration and hence enable higher conductivity [33]. Furthermore, the formation of ion pairs can shift the electrochemical window of the electrolyte and the chemical constitution of the SEI [26, 30]. Recent computational studies show the impact of the solvation structure on the stability of anions in the multivalent electrolytes [30, 34]. The solvation structure of a liquid electrolyte and formation of ion pairs are known to depend on various parameters such as the nature, charge, size of the ionic species, total concentration, the dielectric constant, as well as chelating properties of the solvent, and the temperature [35]. For example, it has been observed that larger cations have a higher tendency to form complexes [36]. Recent simulation studies of multivalent electrolytes also show that the nature and geometry of the solvent molecule also play an important role in determining the association of the ions in solution [30]. Approaches to design electrolytes by gaining an understanding of the solvation structure and ion pairing—and their effect on the stability of inorganic salts and organic solvent, ionic conductivity, viscosity are imperative in building metrics to identify electrolyte components and formulations with better stability, solvation, and conductivity [30]. Considering the crucial role of electrolytes in the development of high-energy-density and safer multivalent batteries, in this review article we aim to provide a comprehensive analysis of the composition and solvation structure, with a particular focus on ion association, and its impact on battery performance for a range of multivalent electrolytes, covering Ca^2+^, Mg^2+^, and Zn^2+^ systems. We also discuss key advancements, major hurdles, and possible future directions in the research of multivalent electrolytes.

## Magnesium Electrolytes

Mg metal possesses a multitude of exceptional properties such as high volumetric capacity (3832 mAh/ml), negative reduction potential (− 2.4 V vs. SHE), low equivalent weight (12.15 g/eq.), low cost (~ $2/kg), high melting point (922.15 K), eco-friendliness, and high abundance in the earth’s crust, making it a good candidate anode material for high-energy–density rechargeable batteries. However, the reductive reactivity of Mg metal with atmospheric gasses such as O_2_, H_2_O, CO_2_, and traditional non-aqueous electrolytes result in the formation of a passivation layer that is both electronically and ionically insulating. Furthermore, in contrast to Li-ion intercalation chemistry, very few solids are known to reversibly intercalate Mg^2+^ ions and no electrolytes have demonstrated the stability and conductivity required to enable a > 4.0-V electrochemical window while exhibiting reversible Mg metal stripping and deposition. Today’s Mg electrolytes are limited by their (1) insufficient anodic stability, which makes them incompatible with high-voltage cathode materials, (2) inability for reversible electrodeposition, and (3) low conductivity. Hence, intense efforts have been devoted to developing novel electrolytes to enable high-energy–density Mg-ion batteries. The high negative potential and activity of Mg metal render aqueous Mg electrolytes impractical, resulting in a preference for non-aqueous Mg electrolyte solutions. The difference in bonding and coordination environment of Mg ions in organomagnesium salts and simple Mg salts such as Mg(TFSI)_2_ and Mg(BH_4_)_2_ impacts the performance. For example, it was observed that the electrolyte exhibiting the highest degree of covalent bonding with Mg^2+^ such as C_2_H_5_MgCl results in the formation of complex species that are capable of reversible electrodeposition at the metal anode. On the other hand, ionically bonded compounds such Mg(ClO_4_)_2_ and Mg(BF_4_)_2_ result in the formation of CIPs and AGGs, which were found to be capable of supporting intercalation in most solvents but fail to show reversible electrodeposition of Mg^2+^ [5]. Gregory suggested that salts with weakly covalent bonding between Mg^2+^ and a bulky anion, such as BBh_2_Ph_2,_ would result in SSIPs or CIPs between covalently bonded complexes and promote both reversible electrodeposition as well as intercalation [5]. Hence, the partial charge on the atoms and steric hindrance of the ligand attached to the metal ions have a significant effect on the interaction between metal cation and ligands, which ultimately control the reversible electrodeposition and intercalation of Mg^2+^ ions.

### Simple Inorganic Mg Salts

**Fig. 1:**
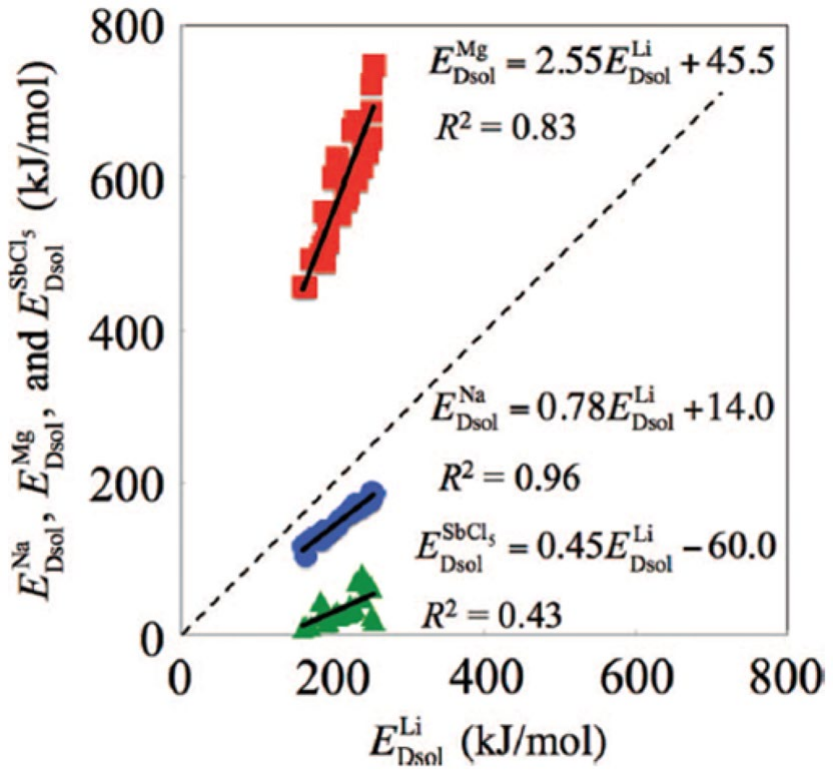
Relationships between de-solvation energies of Li ion and monovalent (Na:* blue circle*) ion and multivalent ion (Mg:* red square*) ions, as well as SbCl_5_:* green triangle*). The* dashed line* corresponds to the energies of Li ion. Republished with permission of Journal of the Electrochemical Society from ref 41; permission conveyed through Copyright Clearance Center, Inc

Conventional simple inorganic Mg salts, such as Mg(BF_4_)_2_, Mg(ClO_4_)_2_, Mg(CF_3_SO_3_)_2_, Mg(SO_4_)_2_, Mg(NO_3_)_2_, MgCl_2_, and Mg(PF_6_)_2_, suffer from poor solubility in most solvents (< 0.5 M in ether) such as esters, ethers, alkyl carbonates, and are found to be incompatible with reversible stripping/plating at the Mg metal anode [5, 37]. Early studies of Mg salts in conventional polar aprotic solvents, namely acetonitrile (AN), propylene carbonate (PC), and tetrahydrofuran (THF), demonstrate the formation of a passivation layer and very high impedance due to either the reduction of solvent molecules (AN, PC) or inactivity of the solvent (THF), which leads to the reduction of salt anions and the deposit of electronically insulting species on the metal surface [37–39]. This passivation film, which is formed as a result of reactions between the active metal and solution species, does not allow conduction of Mg^2+^ ions in contrast to the functional surface films (solid–electrolyte interface, SEI) that form on negative electrodes in commercially available Li-ion batteries. The solvation structure of multivalent ions is likely to play a crucial role in determining both the formation of passivation film at the negative electrode as well as intercalation of ions at the positive electrode. It has been established that divalent ions such as Mg^2+^, Zn^2+^, and Ca^2+^ tend to form more stable ion pairs in solution as compared to monovalent alkali metal ions [40, 41]. A systematic theoretical study of the binding energy between different mono- as well as multivalent ions with a gas-phase solvent molecule by Okoshi et al. [41] shows remarkably larger binding energies for multivalent ions as compared to monovalent ions (Fig. 1). Assuming that higher molecular binding energies correlate with larger de-solvation energies in the liquid phase, it is suggested that the kinetics of charge transfer in multivalent systems would be more sluggish.

**Fig. 2:**
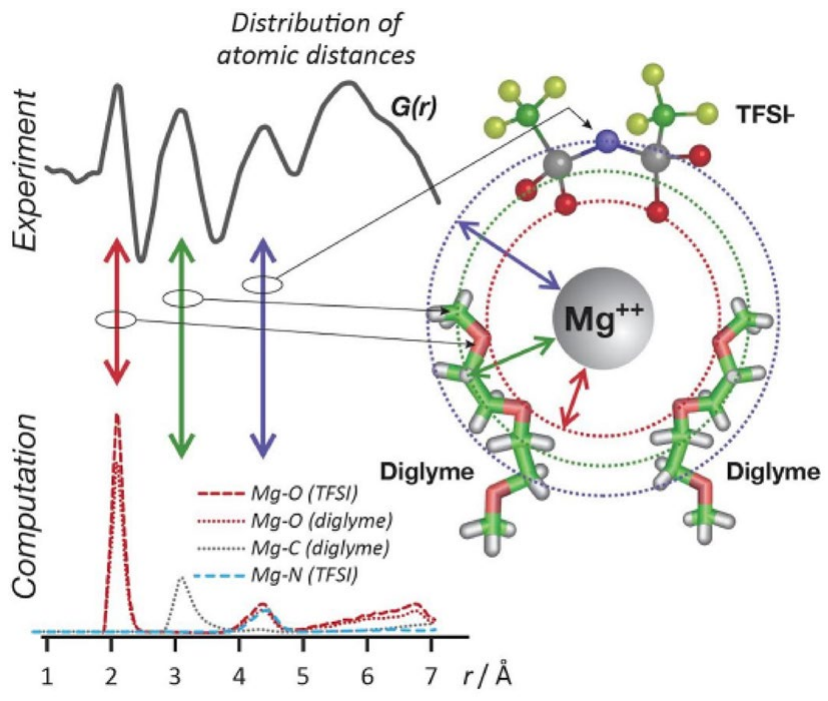
Solvation structure of Mg(TFSI)_2_/diglyme solution from r-weighted form of the d-PDFs, rG(r), highlights the well-defined inner sphere features and broader solvation shells at longer distances (top). Mg-X radial distribution functions from MD simulations (O:* red*, N:* blue*, C:* grey*, S:* yellow*). The corresponding simulation showing Mg^2+^ in space-filling format (*magenta*), TFSI^_^ and diglyme in stick format (*bottom*). Reproduced from Ref. 43 by permission of the PCCP Owner Societies

Even though Mg^2+^ (0.65 Å [42]) exhibits approximately the same size as Li^+^ (0.6 Å [42]), due to high charge density of magnesium, Mg electrolytes are highly prone to ion pair and complex formation, even at modest concentrations for a wide range of solvents [30, 32, 34, 43, 44]. Early work by Minofer et al. using MD simulations reported strong ion pair formation and even clustering in 0.5 M aqueous solution of Mg(OAc)_2_ [6]. However, only recently the detailed solvation structure of non-aqueous Mg electrolytes was explored, where Rajput et al. examined the impact of bulk Mg electrolyte properties on the performance of Mg-ion batteries using a high-throughput computational approach [7, 8]. Mg(TFSI)_2_ is one of the few simple salts known that can be easily dissolved in many organic solvents and ionic liquids and show very high anodic stability. However, its compatibility with the Mg metal anode is still in question and often high overpotential and low coulombic efficiency have been observed for deposition and dissolution [44, 45]. The bulky size and highly delocalized charges of TFSI^−^ typically result in better dissociation and lesser tendency to form ion pairs. Also, the connected* p*-orbitals in the TFSI^−^ anion lower the total energy of the molecule and contributes to its stability. However, an analysis of the solvation structure of Mg(TFSI)_2_ in diglyme using MD simulations and X-ray total scattering by Saul et al. reveal formation of CIPs even at a moderate concentration of 0.4 M, where Mg^2+^ is sixfold coordinated by oxygen atoms including both a TFSI^−^ anion, and diglyme solvent molecules (Fig. 2), to form octahedral or distorted octahedral geometry in solution [43]. Recently, Raman spectroscopy also observed such sixfold coordination of Mg^2+^ ions in Mg(TFSI)_2_/glyme solution in which two ether oxygen atoms originate from monoglyme and four oxygen atoms from the TFSI^−^ anion [43]. However, due to the high flexibility of glymes and different conformers of the TFSI^−^ anion, the number of oxygen atoms donated by glymes and TFSI^−^ anion can vary based on salt concentration. Both simulation and experiment validate that the TFSI^−^ anion coordinates with metal cations (Li^+^, Mg^2+^, Zn^2+^) through the oxygen atoms and not the nitrogen or fluorine [8, 43]. Mg^2+^ is also known to form larger complexes through bridge formation including two or more cations, especially at high concentrations [13, 15]. In electrolytes based on ionic liquids, such as Pyr_14_-TFSI or BMPyr-TFSI with Mg(TFSI)_2_, the Mg^2+^ cation is surrounded by three to four TFSI^−^ anions with more bidentate and bridging TFSI^–^ anions due to a higher population of TFSI^−^ in the solution [44]. Such ion association not only alters the effective charge of the solvated ions ([Mg_n_(TFSI)_m_]^(m−2n)−^) but also affects the dynamics of ionic species in the solution as well as kinetics/energetics for desolvation at an electrode–electrolyte interface [43].

**Fig. 3:**
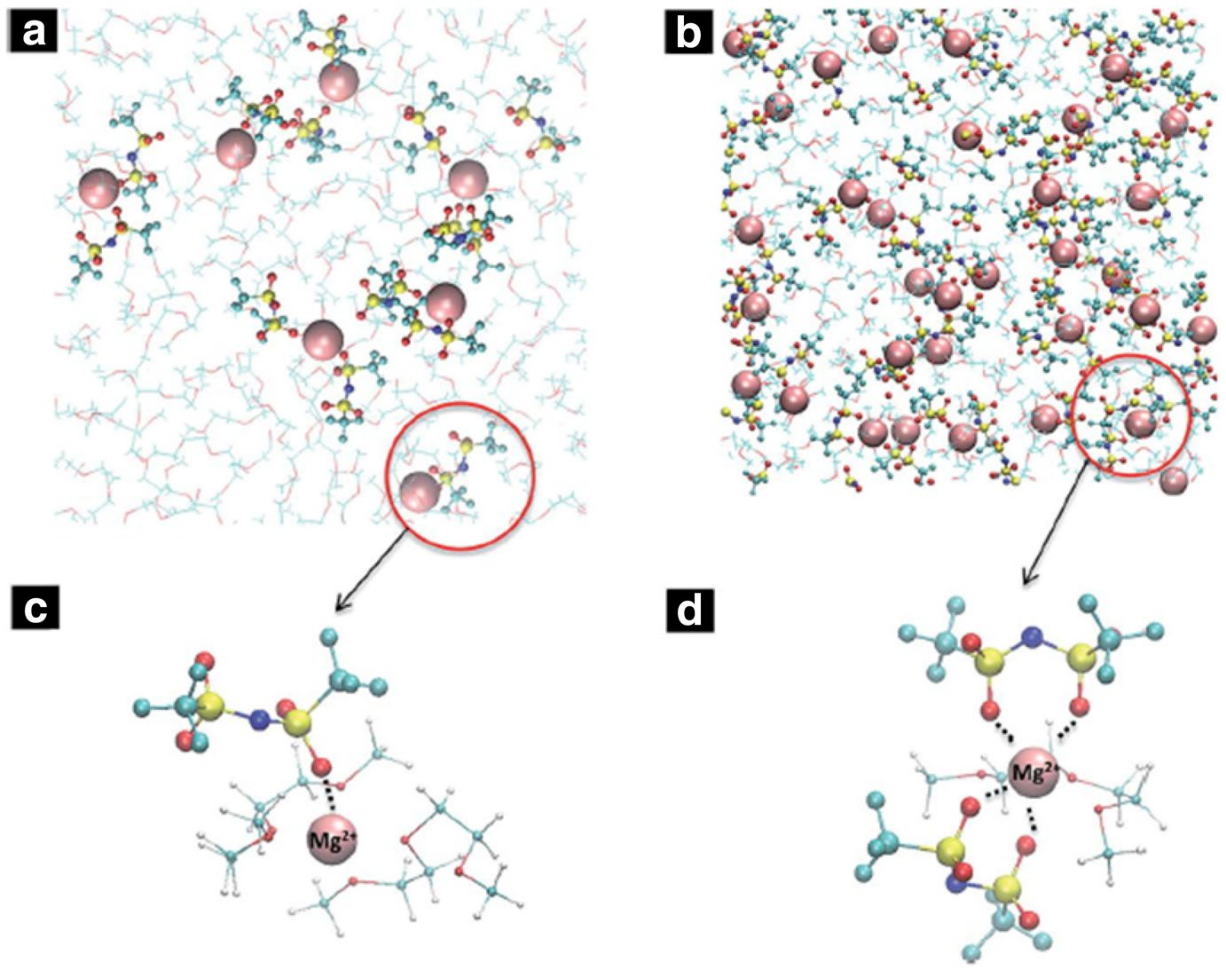
Representative simulation snapshot of** a** 0.1 M;** b** 1.5 M Mg (TFSI)_2_/diglyme at 298 K. Mg depicted in* pink* in space-filling format, TFSI in* licorice*, and diglyme in* line format*;** c** and** d** show the zoomed image of solvation structure around an Mg ion in 0.1 M and 1.5 M concentrations, respectively. Reproduced from Ref. 45 by permission of The Royal Society of Chemistry

The concentration of the solute is a critical parameter in determining the formation of different ionic species in the solution, which in turn control the electrochemical performance of the electrolyte. High-concentration (> 1 M) electrolytes are often preferred, as they can potentially provide high ionic conductivity that in turn lowers the system’s internal resistance [45]. Cyclic voltammograms of Mg(TFSI)_2_/diglyme suggest an increase in current density of magnesium deposition/dissolution with an increase in salt concentration from 0.1 M to 1.0 M and a decrease with further increase in concentration [45]. For Mg(TFSI)_2_/diglyme classical MD simulations show the formation of SSIPs at low concentrations (< 0.4 M) and CIPs at higher concentrations, but no AGGs are observed even at 1.5 M due to the non-coordinating nature of TFSI^−^ (Fig. 3a) [30]. However, higher numbers of monodentate coordination were observed at lower concentrations, whereas TFSI^-^ anions show more bidentate coordination at higher concentrations (Fig. 3b). The latter could significantly increase the desolvation energy, resulting in higher overpotential. Such increase in bidentate coordination, which increases the oxygen atom coordination with Mg^2+^, and CIP formation at higher concentration also negatively affects the ionic conductivity and transference number. Raman spectroscopy studies of the solvation environment of Mg(TFSI)_2_-ionic liquid also suggest the formation of both* cis*- and* trans*- conformer of TFSI^−^ in the solution, with predominantly monodentate interactions between TFSI^−^ and Mg^2+^ at low concentrations and bidentate interactions at high concentrations [46]. Hence, it likely that at low concentrations TFSI^−^ anions which exist as CIPs are in the minimum energy *transoid* (CF_3_ groups on the opposite side of the S–N–S plane) state, while at higher concentrations, a slightly higher local minima *cisoid* (CF_3_ groups on the same side of the S–N–S plane) with ~ 3.5 kJ/mol energy barrier compared to* trans*- conformer exists [28, 46]. Such increase in bi-dentate and bridging TFSI^-^ affects the dynamical properties as well as the desolvation energy of ions in the solution. Different solvates of varying sizes, including free TFSI^-^, CIPs with mono and bidentate coordination and bridging aggregates with several metal centers have also been observed in the solution as a function of concentration [44–46].

The solvation structure depends strongly on the nature of the ligand, its geometry, and the concentration of the solute. Using MD simulations, Rajput et al. reported that for most Mg salts, SSIPs are observed in high-donor-number solvents such as DMSO and in long-chain glymes (e.g., tetraglyme) due to their high oxygen-donor denticity and flexibility to chelate around the Mg^2+^ [30]. Glymes are known to enhance the solvation of metal ions via ion–dipole interaction with the oxygen atoms exhibiting high electron donicity [20]. Kimura et al. [47] also reported formation of SSIPs for Mg(TFSI)_2_/triglyme(G3) by forming an [Mg(G3)_2_]^2+^ cationic species for a concentration range of 0–1.6 M using Raman spectroscopy and DFT at the B3LYP/6-311 + G** level. The strong chelating effect of neutral glyme molecules on metal cations reduces the cation–anion interaction, which promotes SSIPs, as observed previously for Li salts [48]. Contrary to organic electrolytes such as Li salts in PC, Kimura et al. [47] observed an increase in ionicity (dissociativity) for Mg(TFSI)_2_/triglyme electrolyte with an increase in salt concentration. A systematic study by Watanabe et al. using impedance and pulsed-filed gradient spin-echo NMR also reported a similar increase in ionicity for Li(TFSI)_2_/triglyme in concentrated solutions (~ 3 M) [49, 50]. On the other hand, the MD simulations of Mg(TFSI)_2_ in short-chain glymes such as diglyme (G2) and dimethoxyethane (G1) reported formation of CIPs and AGGs, respectively [30]. From both MD simulations and Fourier-transform infrared (FTIR) analysis, it was inferred that the tendency of Mg salts to form ion pairs in glymes monotonically decreases with an increase in chain length of glymes [20, 30, 51]. Similar to Watanabe’s work on Li salt/glyme solution [49, 50], Watkins et al. [46] also reported an alternative class of ionic liquids known as “solvate” ionic liquids for Mg salts/glyme solutions with a general formula of M(Glyme)_m_(X)_n_ [M = Li, Mg; *X* = counter ion]. For a weakly Lewis basis anion such as TFSI^−^, M(Glyme)_m_(TFSI)_n_ solutions behave as a typical ionic liquid with [M(Glyme)_m_]^+^ as the cationic species and exhibit properties similar to those of ionic liquids such as high oxidative stability, high thermal conductivity, high ionic conductivity, etc. [46, 50]. Solvated ionic liquid studies have primarily been performed for Li salts, and obtaining a detailed understanding of the properties of solvated ionic liquids for Mg electrolytes will be extremely beneficial.

**Fig. 4:**
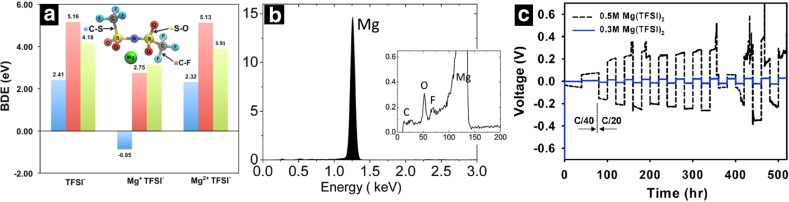
**a** Bond dissociation energy (BDE) of TFSI^−^ anion in different chemical environments corresponding to well-solvated, Mg^1+^ ion paired and Mg^2+^ ion paired configurations from DFT calculations. Adapted with permission from Ref 30. Copyright 2015 American Chemical Society.** b** Energy-dispersive X-ray spectroscopy analysis of deposited Mg metal from a Mg(TFSI)_2_/diglyme electrolyte.* Inset* shows zoomed image at low energy. Reproduced from Ref. 45 by permission of The Royal Society of Chemistry.** c** Galvanostatic cycling of Mg/Mg cells at a rate of C/20 after the first cycle at C/40. Adapted with permission from Ref. 20. Copyright 2014 American Chemical Society

Some widely used solvents such as high dielectric constant nitrogen chelating acetonitrile (AN) (*ε* = 37) and tetrahydrofuran (THF) shows formation of AGGs for most Mg salts resulting in very low solubility [20, 30]. Significant ion pairing of the TFSI^−^ anions with Li^+^ has also been observed in solvents such as glymes and acetonitrile, even at dilute concentrations [28, 52]. However, Raman spectroscopy results show a stronger interaction between Mg-TFSI than Li-TFSI due to the higher charge density and hardness of Mg^2+^ as compared to Li^+^ [46]. The size of the anions and charge distribution also play an important role in forming CIPs and AGGs, for example, the steric effects and delocalization of negative charge in TFSI^-^ anions reduce the tendency to form ion pair compared to the small-sized BH_4_^-^ and BF_4_^-^ anions. Hence, not just the dielectric constant of the solvent but also the size of the solvent and anions, donor number and denticity of solvents, and coordination property of the chelating ligands play a crucial role in determining the speciation of the solution. Even though numerous species have been reported in the literature for simple Mg salts, understanding the relationship between the solvation structure and the electrochemical properties of the electrolytes is still in its infancy. Mohtadi et al. [53] suggested that one possible reason for the failure of salts such as Mg(TFSI)_2_ in organic solvents could be the thermodynamic potential of Mg ion insertion into the host matrices. However, recent computational study suggests that intermediate reduced Mg^1+^ species can activate new decomposition modes for the species coordinated to the Mg ion in an ion paired configuration. Specifically, at Mg metal potentials, the ion pair undergoes partial reduction at the Mg cation center (Mg^2+^ → Mg^1+^), which competes with the charge transfer mechanism and activates the anion to render it susceptible to decomposition, thereby limiting the cathodic stability of the electrolyte (Fig. 4a) [30]. Recent scanning electron microscopy study [45] of Mg(TFSI)_2_/diglyme solution for a concentration range from 0.1 to 1.5 M also observed trace signal of elements C, O, and F, confirming decomposition of anions possibly by initiation with C–S bond breaking or solvent molecules during cycling as observed in simulations (Fig. 4b), [30, 45]. Ha et al. [20] also observed a difference in cathodic stability behavior of Mg(TFSI)_2_/glymes electrolyte as a function of salt concentration (Fig. 4c), which is possibly due to an increase in ion pair formation as a function of increased concentration ultimately leading to enhanced decomposition of TFSI^−^ as mentioned above. Conversely, anions that are known to support reversible plating/stripping of Mg metal, such as BH_4_^−^, exhibit stable bonds even when ion paired with the transient, highly reactive Mg^+^ species [30].

**Fig. 5:**
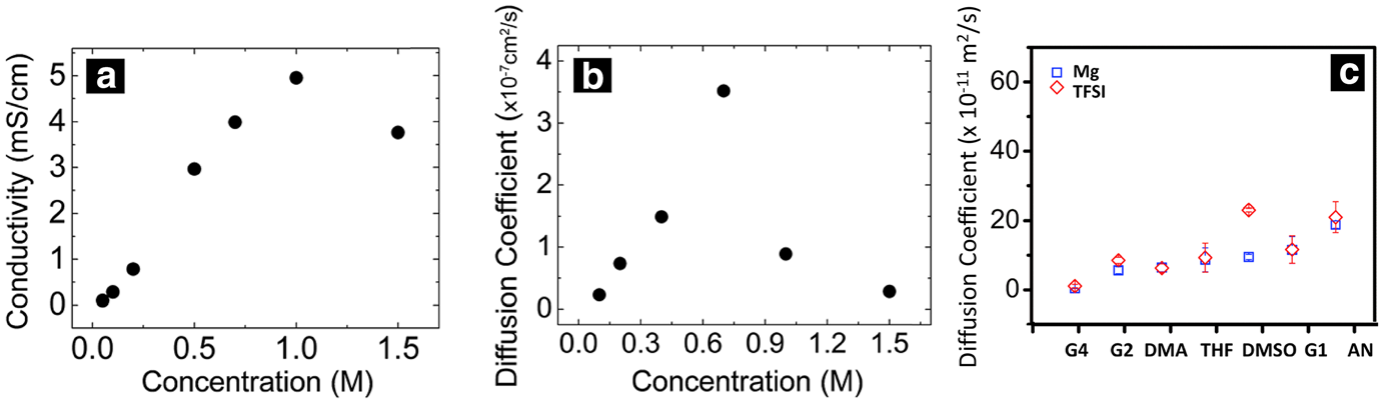
**a** Concentration-dependent ionic conductivity and** b** diffusion coefficient of Mg(TFSI)_2_/diglyme electrolyte at a concentration range from 0.1 to 1.5 M. Reproduced from Ref. 45 by permission of The Royal Society of Chemistry.** c** Self-diffusion coefficients of Mg^2+^ and TFSI^−^ in seven different solvents (*shown on x-axis*) displayed with* error bars*. Adapted with permission from Ref 30. Copyright 2015 American Chemical Society

As discussed above, an increase in salt concentration is often known to lead to the formation of CIPs or AGGs, which in turn decreases the conductivity and increases the viscosity of the solution and is also likely to put a penalty on the stability of the delivery vehicle. It should also be noted that CIPs with multivalent ions, [Mg(TFSI)_*n*_]^(2−*n*)^, are cationic complexes, unlike the neutral CIPs, [Li(TFSI)_*n*_]^(1−*n*)^, formed with monovalent ions. Similarly, the AGGs that form, [Mg_*z*_(TFSI)_*n*_]^(2*z*−*n*)^, can exhibit neutral or anionic composite charges [30]. Hence the effect of CIPs and AGGs on the ionic conductivity and transference number in divalent cation electrolytes are likely quite different than for monovalent systems. At concentrations where CIPs and AGGs form, the conductivity in divalent systems can decrease due to the formation of neutral ion pairs or due to a decrease in the mobility of large charge-carrying species (Fig. 5a, b). Classical MD simulation studies of Mg(TFSI)_2_ demonstrate the formation of AGGs for Mg(TFSI)_2_ in short-chain glymes and acetonitrile and SSIPs in DMSO and tetraglyme, but the diffusion constant of ionic species was found to be faster in AN and short-chain glymes (Fig. 5c) [30]. The faster dynamics observed is likely due to lower viscosity in case of AN and weaker interaction between the ions and the solvent molecules in case of glymes. Hence, interestingly, depending on the properties of solvent molecules and the mechanism of diffusion, it is possible to have a faster mobility of large-size AGGs compared to smaller CIPs.

**Fig. 6:**
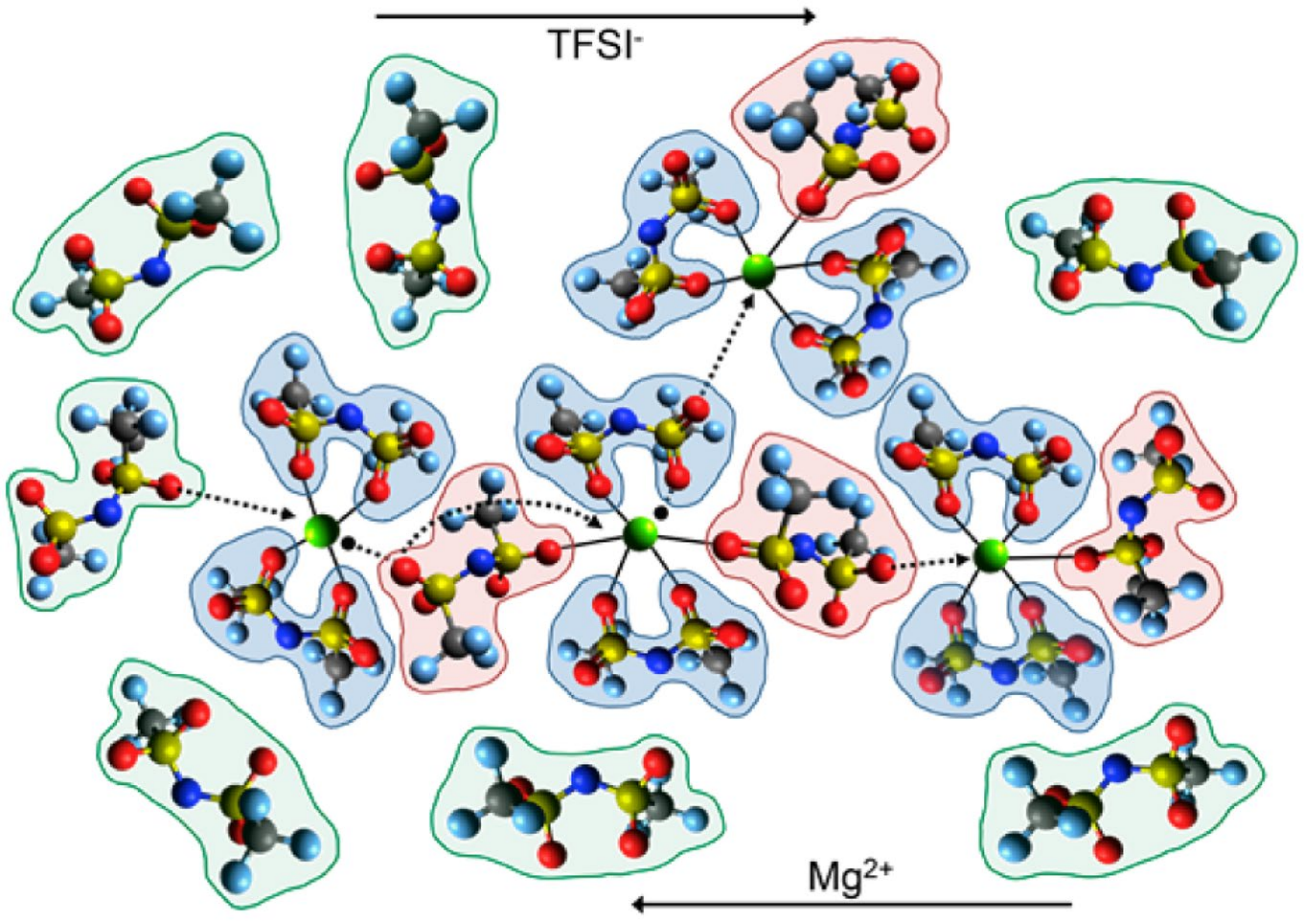
Proposed mechanisms of structural diffusion in the Mg^2+^−IL electrolytes, where the* dashed lines* indicate interactions being formed (▶) and broken (●). The weakly coordinating monodentate and bridging TFSI^−^ anions tend to rapidly exchange between different ionic clusters, resulting in a faster process. On the other hand, strong coordination of bidentate TFSI^−^ anions results in a vehicular diffusion of the cluster followed by a slower process, which involves exchange of the bidentate anions via a bidentate to bridging mechanism. The Pyr1x^+^ cations are omitted for simplicity. Reprinted with permission from Ref 44. Copyright 2014 American Chemical Society

Two modes of ion transport often discussed in the literature are structural and vehicular ion diffusion. Structural diffusion is defined as the exchange of counterion or solvent molecules in the first solvation shell of the metal cation, whereas vehicular diffusion is the motion of metal cation along with the molecules in the first solvation shell. Experimental conductivity measurement of Mg(TFSI)_2_-IL solution observed a decrease in conductivity as a function of salt concentration. Further PFG-NMR results show a decrease in the mobility of TFSI^-^ and an increase in the viscosity of the solution due to increase in Mg^2+^–TFSI^−^ association as the salt concentration increases. Jeremias et al. [44] suggested the possibility of structural diffusion in Mg(TFSI)_2_-IL electrolytes by exchange of TFSI^−^ anions between “free” TFSI^−^ and neighboring [Mg_*n*_(TFSI)_*m*_]^(*m*−2*n*)−^ clusters (Fig. 6). Bidentate TFSI^−^ coordination was found to be stronger and stable as compared to bridge complexes. Hence, due to the strong interaction between bidentate TFSI^−^ anions and Mg^2+^, the mobility of Mg clusters can be higher than the exchange rate of bidentate TFSI- anions. Such hopping of ions explains the non-linear trend of conductivity as a function of concentration observed by Jeremias et al. and the faster diffusion observed for Mg(TFSI)_2_ from MD simulations by Rajput et al. in acetonitrile and monoglyme solutions where AGGs were observed [30]. However, a deeper, detailed understanding of the effect of the molecular organization of the electrolyte and modes of ion transport on the diffusion coefficient, conductivity, and viscosity is desirable.

**Fig. 7:**
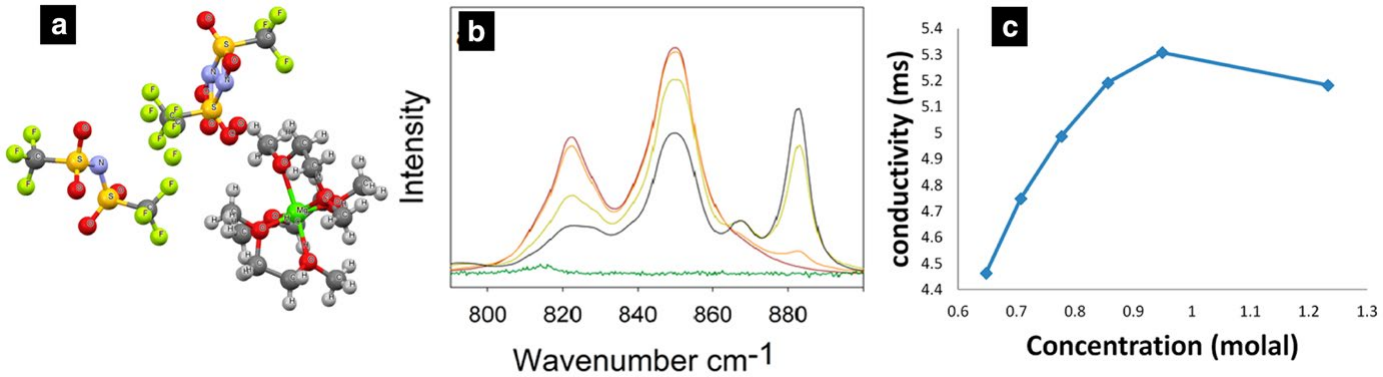
**a** Refined SCXRD structure for MgTFSI_2_ single crystal, recrystallized from MgTFSI_2_/DME solution.** b** Raman spectra of pure DME (*brown line*), U-phase (*orange line*), L-phase (*yellow line*), 1.25 M MgTFSI_2_ solution in DME, and powder pure MgTFSI_2_: 750−900 cm^−1^ spectral region.** c** Conductivity measurements of MgTFSI_2_/DME solutions at various salt concentrations. Adapted with permission from Ref 54. Copyright 2016 American Chemical Society

Salama et al. [54] combined DFT, NMR, and single-crystal X-ray diffraction (SCXRD) to explore the solvation structure of Mg(TFSI)_2_ in dimethoxyethane (DME). DME was found to be a promising solvent in both Li-oxygen and Li-sulfur chemistries and results in higher conductivity of the ionic species, while TFSI^−^ -based salts are highly soluble due the delocalized charge and larger size of the TFSI^−^ anion as discussed above. At all concentrations studied (0.05–1.3 M), the dissolution of Mg(TFSI)_2_ results in Mg.3DME as a stable moiety, where Mg^2+^ ions are encaged by three DME molecules (Fig. 7a). The Raman spectra results for Mg(TFSI)_2_/DME solution indicate the formation of either SSIPs or free TFSI^-^ ions irrespective of the concentration (0.05–1.3 M) (Fig. 7b). These results are in disagreement with the previous simulation and experimental studies of Mg salt in glymes, which suggests the formation of CIPs and AGGs in short-chain glymes such as DME and diglyme [20, 30, 34, 44]. Indeed, the experimental conductivity measurements by Salama et al. show a decrease in conductivity after 0.9 M, indicating the formation of AGGs and increase in viscosity (Fig. 7c). Hence, it is surprising that Salama et al. observed SSIPs and free anions for Mg(TFSI)_2_/DME irrespective of the salt concentration. Even though spectroscopy techniques such as NMR and Raman are powerful techniques, they have been reported to be inadequate to provide reliable information about ion association equilibria [55]. As the detection limit of NMR [1] H and [13] C is typically low, it is possible that some minority species were not detected by the NMR studies performed by Salama et al.

**Fig. 8:**
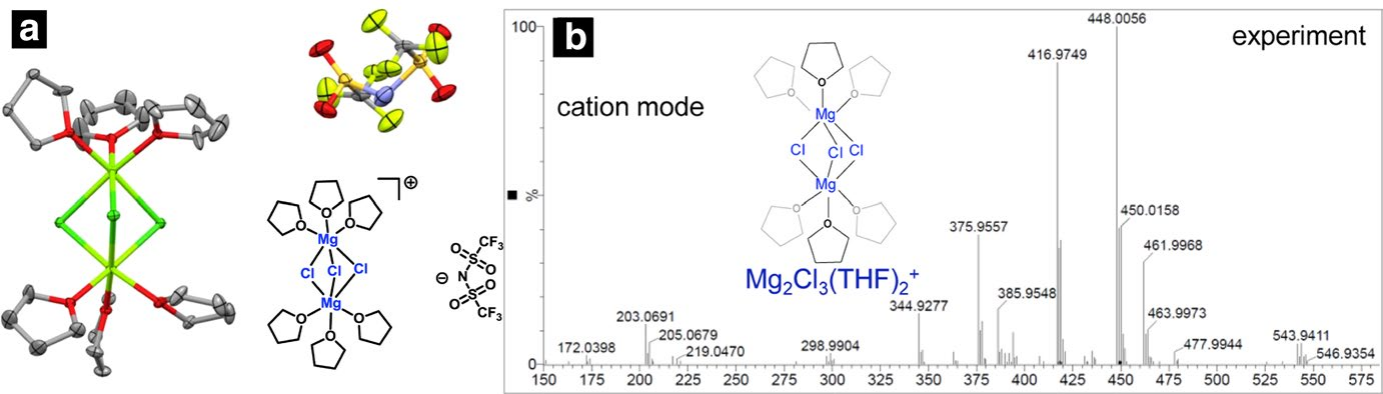
**a** Displacement of ellipsoid representation of [Mg_2_Cl_3_(THF)_6_]^+^[TFSI]^−^.** b** ESI–MS spectrum of 0.5 M Mg(TFSI)_2_-MgCl_2_ at the ratio of 1:(0.5) in THF (experimental measurement in positive mode). Adapted with permission from Ref 56. Copyright 2016 American Chemical Society

Several recent studies have reported enhancement in electrochemical performance through the current density and coulombic efficiency for reversible Mg deposition with the addition of MgCl_2_ to Mg salts such as Mg(TFSI)_2_ [56]. Although, while a detailed understanding of the species present in the solution in the mixed salt system is pending, a single-crystal diffraction and electrospray ionization mass spectroscopy (ESI–MS) study by Sa et al. reveal formation of an ion pair complex composed of a cation [Mg_2_Cl_3_(THF)_6_]^+^ and the [TFSI]^_^ anion (Fig. 8) in the solution [56]. The cationic species reported here is the widely accepted electroactive species present in the magnesium organohaloaluminate or organomagnesium electrolytes in THF responsible for the reversible magnesium deposition/dissolution. This suggests that irrespective of anions such as TFSI^−^, magnesium and chlorine have a strong tendency to form a bridge complex where chloride ions are shared by more than one magnesium ion. As previous simulation results suggested formation of AGGs in Mg(TFSI)_2_/THF solution [30], it is likely that the addition of MgCl_2_ results in a competing interaction between TFSI^–^ and Cl^–^ anions, where the strong electrostatic interactions between Mg^2+^-Cl^−^ results in formation of a cationic complex species while the weaker ionic interaction between Mg^2+^-TFSI^-^ results in formation of CIPs of TFSI^-^ anion with the [Mg_x_Cl_y_]^+^ complex.

**Fig. 9:**
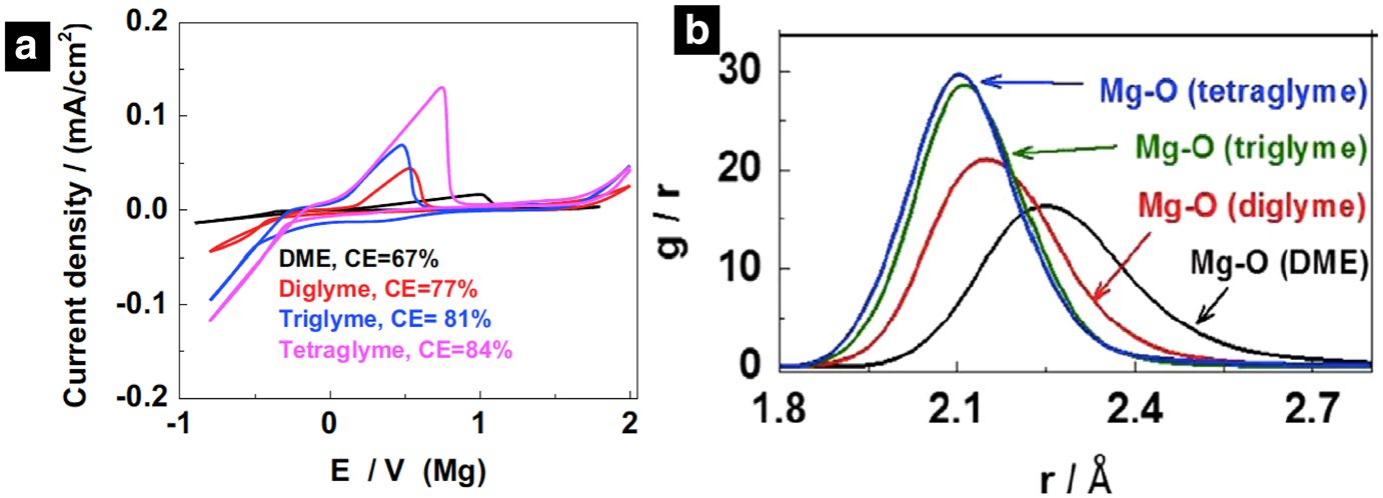
**a** Cyclic voltammograms (20 mVs^_1^) of 0.01 M Mg(BH_4_)_2_ in glymes on a Pt electrode.** b** Radial distribution function of Mg–O(solvent) of Mg(BH_4_)_2_ in DME, diglyme, triglyme, and tetraglyme from MD simulations. Reprinted from Ref. 51 with permission from Elsevier

Mg(BH_4_)_2_ is an example of another simple and non-corrosive inorganic Mg salt that has gained much popularity over the last few years due to its compatibility with a Mg metal anode [57, 58]. Both Mohtadi et al. [53] and Shao et al. [57] reported significant ion pair and aggregate formation of Mg(BH_4_)_2_ in diglyme, dimethoxyethane, and THF solvents using IR and NMR spectroscopy analyses [53, 57]. Later MD simulations confirmed a strong tendency of Mg(BH_4_)_2_ to form CIPs and AGGs in most solvents except DMSO and tetraglyme [30]. Also, stronger interaction between Mg^2+^ and BH_4_^-^ was observed in THF as compared to DME in agreement with previous experimental results reported by Mohtadi et al. The interaction between Mg^2+^ and BH_4_^-^ was observed to be mediated through the Mg-H bond instead of the Mg-B bond, which results in a strong covalent bonding between Mg^2+^-BH_4_^-^, especially in weakly coordinating solvents [51, 57, 58]. Yuyan et al. investigated the effect of ligands on the electrochemical properties of Mg (BH_4_)_2_/glyme electrolytes [51]. They observed that the electrochemical performance (coulombic efficiency, overpotential, and current density) of Mg(BH_4_)_2_ is enhanced with an increase in the chain length of glyme from DME (G1) to tetraglyme (G4) (Fig. 9a). Classical MD simulations demonstrate that the Mg–O(Glyme) distance decreases and the coordination number of Mg–BH_4_ decreases monotonically with an increase in chain length of glymes from DME to tetraglyme, indicating stronger coordination between Mg^2+^ and longer chain glymes (Fig. 9b). Similar trends of decreasing cation–anion coordination number with an increase in chain length of glymes were also reported for other Mg salts using MD simulations [30]. It should be noted that the donor number (DN), which is commonly referred to as an index of the Lewis base character of solvent, follows the order DME/monoglyme (24.0) > diglyme (19.5) > triglyme (-) > tetraglyme (16.6) [20] while their dielectric constant is very similar; approximately 7. However, the high oxygen donor denticity and flexibility with an increase in chain length improves the solvation of the metal cation with an increase in chain length. On the other hand, such improved solvation comes at the cost of slow mobility of ionic species, as suggested by the diffusion coefficient results from MD simulation, showing that the mobility of both cation and anion decreases monotonically with an increase in chain length of glymes [30]. Hence, a mixture of glymes is often used to obtain an optimal combination of dissociation and conductivity.

**Fig. 10:**
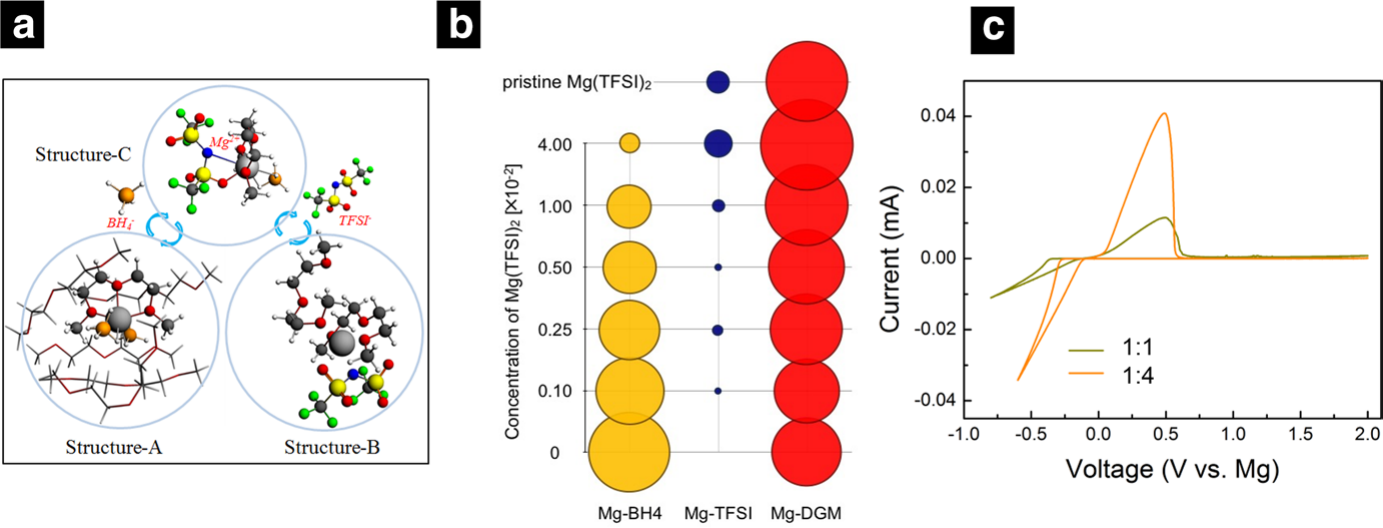
**a** The exchange mechanisms and solvation structures in the combined electrolyte containing both Mg(BH_4_)_2_ and Mg(TFSI)_2_ in diglyme (DGM), highlighting that Structure-A is changed to Structure-B via intermediate Structure-C.** b** Coordination number of Mg-BH_4_, Mg-TFSI and Mg-DGM in mixed solutions of Mg(BH_4_)_2_ and Mg(TFSI)_2_ (**b**) cyclic voltammetry of electrolyte solutions prepared in diglyme with different concentrations of Mg(BH⁠4) 2 and Mg(TFSI) 2 as labeled. The scan rate was 20 mV/s. Reprinted from Ref. 60 with permission from Nano Energy

Few studies have suggested that the addition of additives such as LiBH_4_ or other Mg salts reduces the strong interaction between Mg^2+^ and BH_4_^−^, resulting in an enhancement of the electrochemical performance. However, it is worth noticing that any addition of other electrochemically active species such as Li convolutes the evaluation of the role of the Mg electrochemical response [59]. Recently, Hu et al. investigated the solvation structures and dynamics of Mg(BH_4_)_2_ and Mg(TFSI)_2_ dissolved in diglyme (DGM) at various concentrations and ratios of Mg(BH_4_)_2_/Mg(TFSI)_2_ using a combination of natural abundance ^25^Mg NMR, quantum chemistry calculations of ^25^Mg NMR chemical shifts, classical MD calculations, and electrochemical performance tests [60]. They observed that for 0.01 M Mg(BH_4_)_2_ (which is the saturated concentration in DGM), the first solvation shell of a Mg^2+^ ion contains two BH_4_^−^ anions and one DGM molecule as a tridentate chelating the Mg^2+^, while the second solvation shell consists of five DGM molecules (Fig. 10a Structure-A). In contrast, for the system of Mg(TFSI)_2_ in DGM, at dilute concentrations, TFSI^-^ is fully dissociated from Mg^2+^ while at high concentration Mg^2+^ and TFSI^-^ are only partially dissociated with CIPs formed between Mg^2+^ and TFSI^−^ (Fig. 10a Structure-B). An exchange mechanism between solvation structures in the combined electrolyte containing both Mg(BH_4_)_2_ and Mg(TFSI)_2_ in DGM was found to result in a single observed ^25^Mg NMR peak. Such solvent exchange is responsible for the more uncoordinated anions, improved stability, and ionic conductivity of the mixed anion composition as compared to each single anion solution. For the solvent exchange mechanism, an intermediate Structure-C (Fig. 10a) with its first solvation shell similar to Structure-A but with one BH_4_^-^ replaced by a TFSI^-^ anion was found to be responsible for facilitating the process. Such stable Mg species in mixed Mg electrolytes [Mg-BH_4_-TFSI]/solvent, potentially reduce the possibility of the TFSI^-^ anion decomposition that was observed in Mg(TFSI)_2_/DGM solutions in previous simulation results. By mixing two competing Mg salts, they were able to reduce the strong covalent interactions between Mg^2+^ and BH_4_^−^ anions. A small increase is observed in the coordination number of Mg-TFSI and a significant increase in the interaction of Mg^2+^ ions with glymes (Fig. 10b). The weakest interaction between Mg^2+^ ions with BH_4_^−^ and TFSI^−^ anions were observed when the ratio of Mg(BH_4_)_2_ and Mg(TFSI)_2_ is 1:4. Battery performance tests indicated that the efficiency of reversible plating/stripping of Mg strongly depends on the concentration and the ratios of Mg(BH_4_)_2_ and Mg(TFSI)_2_ in DGM that is optimal at the Mg(BH_4_)_2_ and Mg(TFSI)_2_ ratio of approximately 1:4, owing to both the enhanced molecular dynamics and the stability of the TFSI^−^ anion (Fig. 10c).

Excitingly, Mohtadi et al. recently introduced 3-D boron clusters as potential anions for Mg batteries. Monocarborane, CB_11_H_12_^−^, in an Mg(CB_11_H_12_)/tetraglyme electrolyte, was reported to be compatible with Mg metal and possess a high anodic stability (3.8 V vs. Mg/Mg^2+^) as well as relatively high conductivity (1.8 mS cm^−1^), marking a significant development in practical electrolytes for Mg batteries [61]. In the same study, X-ray diffraction analyses on crystallized Mg(CB_11_H_12_)_2_/dimethoxyethane and Mg(CB_11_H_12_)_2_/diglyme solutions showed the presence of SSIPs containing Mg^2+^ cations bound to solvent molecules in a hexacoordinate configuration. Following Mohtadi’s work, McArthur et al. [62] reported a smaller ten-vertex carborane anion-based Mg salt, [Mg^2+^][HCB_9_H_9_^1−^]_2_ which form SSIPs as observed from ^11^B NMR spectrum and X-ray diffraction study with three DME molecules coordinated with Mg^2+^ in the solid state. Monocarborane is known to be chemically inert and weakly coordinating [61, 63], but further detailed examination of the solvation structure involving this salt from e.g., MD simulations or first-principles calculations is not yet available. Crystal structures and solution NMR data of various carborane salts show the preferred site of interaction for cations at boron atoms opposite the carbon atom of the anion, despite the carbon calculated to be the most negatively charged atom of the cage [64].

### Organometallic Compounds (Complex Salts)

**Fig. 11:**
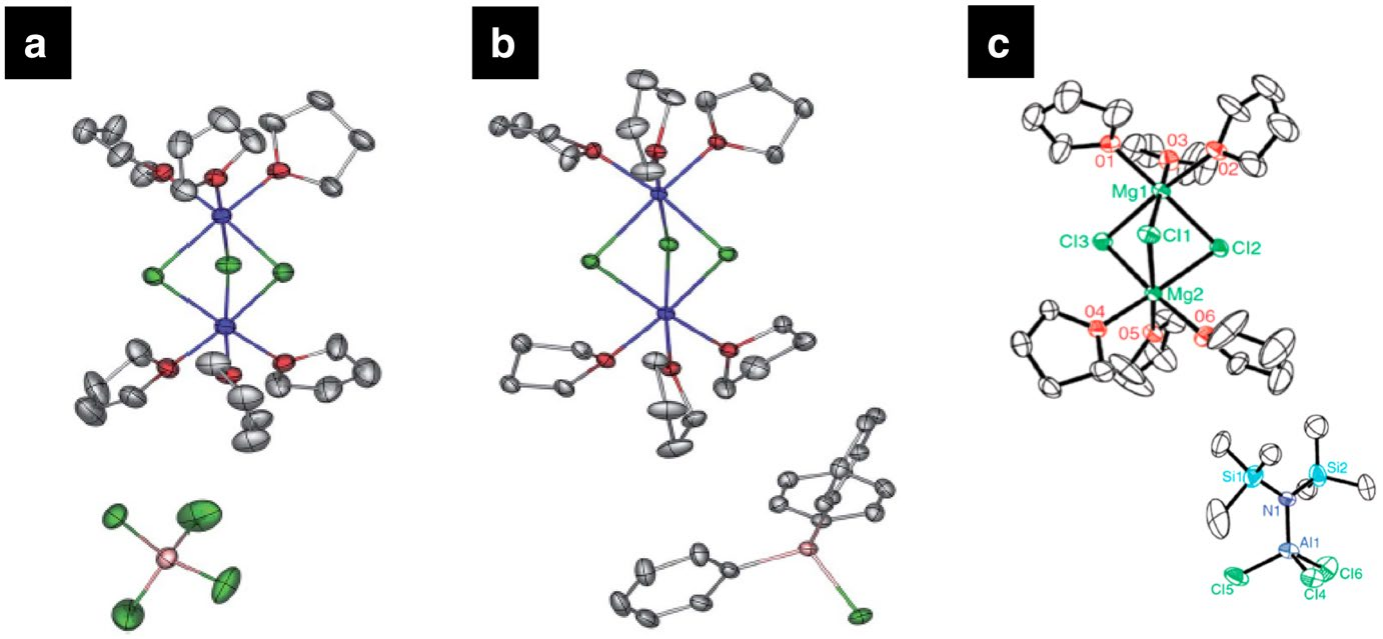
**a** ORTEP plot of [(μ-Cl)_3_Mg_2_(THF)_6_]AlCl_4_.** b** [(μ-Cl)_3_Mg_2_(THF)_6_]AlPh_3_Cl [(μ-Cl)_3_Mg_2_(THF)_6_]AlCl_4_ (Mg,* blue*; Cl,* green*; Al,* pink*; C,* gray*; O,* red*). Reproduced from Ref. 75 with permission from The Royal Society of Chemistry.** c** ORTEP plot (25% thermal probability ellipsoids) of (Mg_2_(μ- Cl)_3_6THF) (HMDSAlCl_3_). Reproduced from Ref. 67 with permission from The Royal Society of Chemistry

Organometallic complex electrolytes in ether solvents comprise some of the few Mg electrolytes known to demonstrate highly reversible electrodeposition due to the stability of the ethers, the RMgX compound, and the Mg(AR_2_R’_2_)_2_ with respect to the metal anode. These electrolytes are highly corrosive [65–67] and the metal deposition process is found to occur by complex adsorption phenomena. The first evidence demonstrating reversible electrodeposition of magnesium from solutions of Grignard reagents in ether solvents was provided by Gaddum back in 1927 [68]. Unfortunately, despite the high stability of organo-magnesium salts against the metallic magnesium surface, the strong reducing character, extremely poor ionic conductivity (few μS/cm), and insufficient anodic stability (< 1 V) curbed their practical applicability in Mg batteries [19, 65, 66]. Through the seminal work of Gregory et al., it was established that the addition of electron-withdrawing Lewis acids such as aluminum chlorides (AlCl_3_) with Lewis bases such as Grignard reagents (RMgCl) and dibutylmagnesium (Bu_2_Mg) can significantly enhance the oxidative stability by stabilizing the R-Mg bond in Grignard solutions [5]. In the last three decades, there have been continuous efforts in enhancing the anodic stability and conductivity of Grignard solutions by Lewis acid (aluminum or organoboron) neutralization while at the same time developing suitable cathode materials that can intercalate Mg ions in nonaqueous media. A breakthrough was made in 2000 when Aurbach et al. demonstrated the first successful prototype of a rechargeable Mg battery consisting of an Mg anode, a Chevrel phase molybdenum sulfide (Mo_6_S_8_) cathode, and a magnesium organohaloaluminate electrolyte solution. From meticulous screening of several electrolytes synthesized by the acid–base (R_*m*_MgCl_2−*m*_–R′_*n*_AlCl_3−*n*_) reactions, a complex obtained from the reaction of EtAlCl_2_ and Bu_2_Mg with a molar ratio of 2:1, namely the dichloro complex (DCC) dissolved in THF exhibited the highest anodic stability (~ 2.4 V vs. Mg/Mg^2+^), an impressive cycle life (> 2000 cycles), improved ionic conductivity of 1.4 mS/cm and an energy density of ~ 60 Wh/kg [19]. The question of which electroactive species governs the enhanced oxidative stability and highly reversible Mg deposition/dissolution in Grignard electrolytes has intrigued the scientific community. Figure 11a shows the widely accepted single-crystal structure observed in the DCC electrolytes, which was obtained by the addition of a nonpolar cosolvent such as hexane or by precipitation at low temperature [65, 66]. It was observed that Grignard solutions are composed of chloride-bridged species, where the active cation may include more than one Mg ion and exhibit a general structure of Mg_2_R_3−*n*_Cl_*n*_^+^ROR while the aluminium-chloro-organo anions likely exhibit the general structure of AlCl_4−*n*_R_*n*_^−^ [66, 69]. However, the accurate structures of the different electroactive species present in the solution in dynamic multiple equilibria as a result of transmetallation reaction cannot be revealed by crystallographic analysis [66]. The structure of the cationic and anionic species in the complex solution is dependent on the nature and ratio of the Lewis acid and base, the solvent, concentration, temperature, and nature of the ligand. For example, the electrochemical window of DCC electrolytes is known to be governed by the Lewis acidity of aluminum compounds and complex ionic species formed in the solution [70]. A comprehensive understanding of the effect of different parameters, such as acid–base ratio and nature of ligands on the formation of species in the solution, which controls the chemical and electrochemical properties, is found in the review article by Yoo et al. [69]. Most literature work on Mg organometallic salts refers to the Mg solvates as complexes and not ion pairs, possibly due to high tendency of the Cl^−^ anion to donate a lone pair electron and form a chemical bond with Mg^2+^. Even though we have been unable to locate detailed analyses to confirm if the ligands are present in the inner or outer sphere of Mg^2+^, we refer to Mg species in organometallic solutions as complexes, consistent with available literature. Conversely, when a cation and anion complex is bound through electrostatic interaction, they are defined as an ion pair complex. NMR analysis by Gizbar et al. identified charged complexes formed by chlorine bridges, [Et_2_ClAl-Cl-AlClEt_2_]^−^ and [MgCl]^+^ as the possible electroactive species present in the DCC electrolytes [71]. They suggested that the high conductivity observed at 2:1 acid:base ratio is a result of the formation of charged complexes [Et_2_ClAl-Cl-AlClEt_2_]^−^ and [MgCl]^+^, while neutral complexes [MgCl_2_] formed at 1:1 ratio decrease the conductivity of the electrolyte solution. Many experimental studies including Raman spectroscopy, nuclear magnetic resonance (NMR) in solution phase, and X-ray diffraction (XRD) on the crystallized samples, Mg K-edge near X-ray absorption fine structure (NEXAFS) identified various species in multiple equilibria. These species include charged complexes such as [Mg_2_Cl_3_]^+^ and [MgCl]^+^ as the most general cation species where the inorganic ligand bonds directly to Mg and Mg is always found to be a hexa-coordinated. The majority anion species are of the form AlCl_4−*n*_R_*n*_^−^ (1 ≤ *n* ≤ 3) and exhibit organic ligands which reside primarily bound to Al with tetrahedral coordination. Finally, neutral complexes, MgCl_2_ and AlCl_3−*n*_R_*n*_ (1 ≤ *n* ≤ 3), are also evidenced [69, 71–73]. It is often suggested that the dimer cation [Mg_2_Cl_3_]^+^ exist in the solution through an equilibrium between MgCl^+^, and MgCl_2_. DFT calculations suggest that since the charged complex [AlCl_4_]^−^ possess a low-lying HOMO frontier orbitals, the ion pair complex between [Mg_2_(μ-Cl_3_)THF_6_]^+^ and [AlCl_4_]^−^ should have high anodic stability [67, 74]. The cationic complex [Mg_2_(μ-Cl_3_)THF_6_]^+^ adopts a pseudo-D_3h_ symmetry where two magnesium atoms share three chlorine atoms and each magnesium has three THF ligands attached (Fig. 11a) [75].

**Fig. 12:**
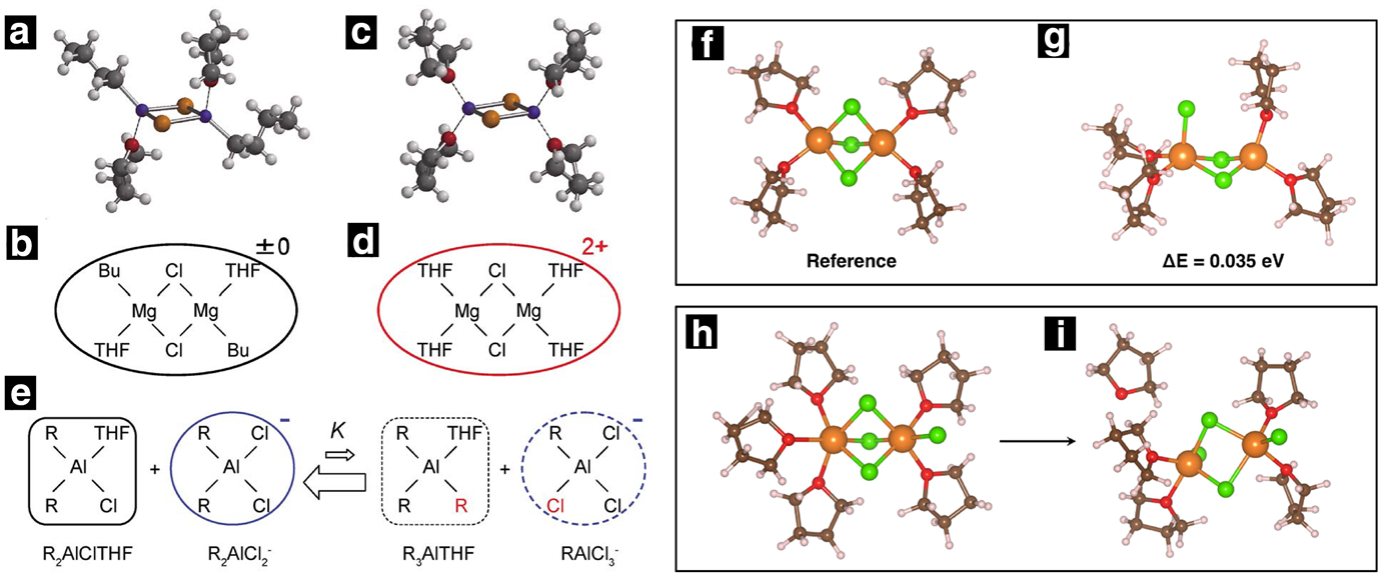
Complex structures of Mg electrolytes.** a**, b Tetrahedral dimer structure of neutral Mg complex in 0.25 M BuMgCl/THF: Mg_2_Cl_2_Bu_2_THF_2_.** c**,** d** Tetrahedral dimer structure of dicationic Mg complex in 0.25 M MgAlCl_2_EtBu_2_/THF: Mg_2_Cl_2_THF_4_^2+^.** e** Tetrahedral monomer structures of Al complexes in 0.25 M MgAlCl_2_EtBu_2_/THF, and their equilibrium, where R = Et or Bu. Republished with permission of Journal of the Electrochemical Society from Ref 70; permission conveyed through Copyright Clearance Center, Inc. DFT-PBE optimized solvation structures for Mg_2_Cl^3+^·4THF in approximate (**f**) C_3v_ and (**g**) D_2h_ symmetry and Mg_2_Cl_4_·5THF in approximate (**h**) C_3v_ and (**i**) D_2h_ symmetry. Reprinted with permission from Ref 76. Copyright 2014 American Chemical Society

**Fig. 13:**
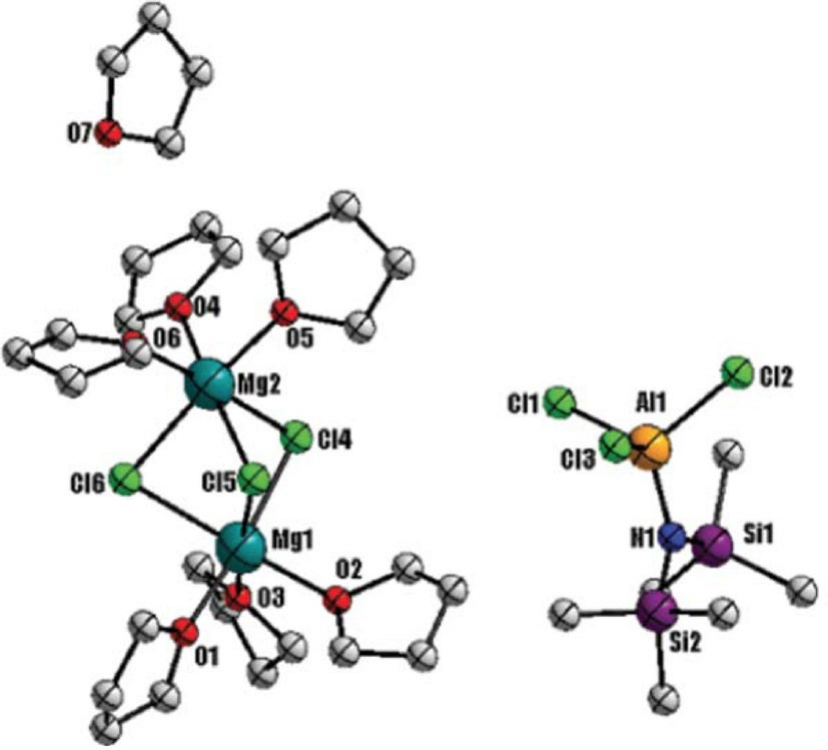
ORTEP plot (50% thermal probability ellipsoids) of [Mg_2_(μ -Cl)_3_THF_6_][HMDSAlCl_3_]THF. Hydrogen atoms are omitted for clarity. Reproduced from Ref. 86 with permission from The Royal Society of Chemistry

Recently, Liu et al. suggested a simple synthesis to form cationic complexes Mg_2_Cl_3_^+^ and MgCl^+^ in solution by using MgCl_2_ as a non-nucleophilic source of Mg^2+^ with an aluminum Lewis acid such as (AlEtCl_2_, AlPh_3_, and AlCl_3_) (Fig. 11a, b) [75]. They provided the first evidence of the THF-solvated MgCl^+^ complex present in the solution using SPIN-MS as traditionally used ESI–MS fails to detect weaker interactions such as Mg-THF [73]. However, contradictory results have been reported in the literature about the species present in solution and the exact solvation structure of Mg ions. Most experimental and simulation studies suggest hexa-coordinated Mg ions, whereas a few experimental studies using NEXAFS and Fourier-transformed extended X-ray absorption fine structure (EXAFS) and first-principle-based simulations reported a tetrahedral coordination for Mg^2+^ ions [70, 76]. A detailed study of the solvation structure of ionic species present in DCC solution was performed by Nakayama et al. [70] using X-ray absorption fine structure (XAFS) and Fourier-transformed extended X-ray absorption fine structure (EXAFS). By comparing the coordination environment of Mg(AlCl_2_EtBu)_2_/THF with BuMgCl/THF and Mg(ClO_4_)_2_/H_2_O, it was concluded that the coordination number of Mg and Al are 2/3 in Mg(AlCl_2_EtBu)_2_/THF and BuMgCl/THF compared to those in aqueous Mg(ClO_4_)_2_/H_2_O and Al(NO_3_)_2_ solutions. The presence of second and third solvation shell for Mg indicated the formation of oligomers while monomers were observed for Al complexes. Contrary to previous reports, they observed tetrahedral coordination for both Mg and Al, where the coordination number of Al varies between 4 and 6 depending on the pH of the solution within the range of 3 < pH < 7 (Fig. 12a). They reported (Mg_2_Cl_2_THF_4_)^2+^, (R_2_AlCl_2_)^−^, and (R_2_AlCl_3_)^−^ as the active ionic species present in the solution responsible for the electrochemical performance of the DCC electrolytes. The first-principle molecular dynamics (FPMD) simulations based studies using DFT (PBE-GGA) by Wan et al. also found the sixfold coordination Mg structures energetically unstable (Fig. 13b below) [76]. They reported tetrahedral coordination of Mg and occasional observation of fivefold coordination in the cationic complexes often known as dimer structures in the electrolyte. They concluded that six-fold Mg species can only be established in the solid phase. However, as previously noted, this is clearly not the case for aqueous solutions. Furthermore, classical molecular dynamics simulations results by Wan et al. using optimized potential for liquid simulations (OPLS) force field parameters failed to reproduce the tetrahedral solvation structure and instead demonstrated sixfold coordination. Cheng et al. pointed out the importance of the nature of the solvent in determining the solvation structure. Using Raman spectroscopy, NMR and single-crystal XRD, they identified a tetra-coordinated doubly charged cation complex, [Mg_2_(μ-Cl)_2_DME_4_]^2+^ when dimethoxyethane (DME) was used as a solvent instead of THF for which they observed six-coordinated cation complex, [Mg_2_(μ-Cl)_3_THF_6_]^+^ [77]. The tetra-coordinated cation complex with DME solvent was found to be highly active for reversible Mg electrodeposition. They suggested that the six-coordinated complexes such as MgCl^+^ and Mg_2_Cl_3_^+^ are unlikely to form because DME is sterically unable to fulfill the coordination number of six. However, in contrast—six-coordination of Mg^2+^ in simple inorganic salts such Mg(TFSI)_2_ has been observed previously in DME solution using molecular simulations [30].

The electrochemical window of the DCC electrolyte was still narrow and limited by the relatively weak Al-C bond, which breaks through a β-H elimination reaction [65, 78]. To overcome the problems of DCC electrolytes, Mizrahi et al. [79] developed the all-phenyl complex (APC) electrolyte solutions by replacing the alkyl ligands with phenyl. By using phenyl as organic ligands, an enhanced ESW of 3.3 V vs. Mg on a Pt working electrode, low overpotential, 100% cycling efficiency, and specific conductivity of  ~ 2–5 mS/cm was achieved. Raman spectroscopy, together with DFT, NMR, and single-crystal XRD suggested Mg_*x*_Cl_y_^+^ (MgCl^+^, Mg_2_Cl_3_^+^, MgCl_2_) as the majority Mg species, where Mg is always six-coordinated and AlCl_4−*n*_Ph_*n*_^−^ (*n* = 0–4) and Ph_4_Al^−^ as the major anionic species features consistently tetra-coordinated Al [74, 79]. Neutral complexes, such as MgCl_2_, were not observed for the best-performing electrolyte with 2:1 ratio of PhMgCl and AlCl_3_, which results in better ionic conductivity [79]. Some air-sensitive nucleophilic species such as Ph_2_Mg and PhMgCl along with minor ratios of PhAlCl_2_, Ph_4_Al^_^, AlCl_4_^−^ were also predicted to be present in the solution. In both DCC and APC electrolytes, ether solvents are part of the actual solvation structure and play an important role in stabilizing the ionic species, but APC electrolytes form a variety of aluminum compounds unlike DCC electrolytes. Early studies reported that the highly nucleophilic and corrosive nature of APC solutions makes them incompatible with electrophilic cathode materials (such as sulfur and oxygen) and electrophilic solvents (such as esters and carbonates) and also prohibits their use with aluminum current collectors [21, 67]. However, full operation of APC electrolytes with Chevrel phase-Mo_6_S_8_, TiS_2_, and V_2_O_5_ cathodes has already been demonstrated by adding either LiCl or MgCl_2_ as additives [80, 81]. Pan et al. demonstrated enhanced electrochemical performance for APC salts in Mg-Mo_6_S_8_ with the addition of MgCl_2_ salt and they observed a typical dimer structure Mg_2_(μ-Cl_3_) from single-crystal X-ray diffraction [82]. Hence, it is likely that addition of MgCl_2_ does not change the active species of APC salts but rather drive the Schlenk equilibrium to generate more electroactive species. However, the addition of MgCl_2_ is likely to make APC salts more corrosive [54, 83]. To enhance air and moisture stability of the Lewis bases, a few groups suggested replacement of (R = Ph) with (R = OPh) and observed complex ion pair formation between [Mg_2_Cl_3_]^+^ and [Al(OR)_4_]^−,^ but these electrolytes still exhibit a highly corrosive nature due to the high chlorine content [84, 85]. Nelson et al. further tried to enhance the performance by using the Lewis acid Al(OPh)_3_ to reduce the chlorine content. Using NMR and ESI–MS, they suggested two distinct active Mg^2+^ charged complexes in solution, [Mg_2_Cl_3_(THF)_4_]^+^ and [Mg_2_(OPh_3_)Cl(THF)_2_]^+^, where magnesium is five-coordinated. The anion complex was identified as [Al(Ph)_4_]^-^ with Al in tetrahedral coordination.

In 2011, Kim et al. proposed a strategy using non-nucleophilic Hauser bases salt comprising hexamethyldisilazide magnesium chloride (HMDSMgCl) electrolyte which demonstrates good compatibility with the electrophilic sulfur cathode [21]. Similar to DCC and APC the crystal structure of HMDSMgCl electrolytes show a typical dimer cation complex [Mg(μ − Cl)_3_THF_6_]^+^ and [HMDSAlCl_3_]^−^ as the anion complex (Fig. 11c). The cationic species observed from single-crystal X-ray diffraction in DCC, APC, and HMDSMgCl in THF show a dimeric magnesium complex, where two magnesium ions share three chlorine atoms forming a bridge complex and each magnesium ion is solvated by three THF molecules [86]. Mg was found to be hexa-coordinated and the counter-anion Al species as tetra-coordinated in all three electrolytes in a majority of experimental studies. Zhao-Karger et al. confirmed the crystal structure of [HMDSMgCl] observed by Kim et al. using NMR for the ratio of 1:2 for HMDSMgCl and AlCl_3_, while other ratios studied from NMR did not yield the same crystal structure (Fig. 13) [86]. Recently, Pan et al. demonstrated that addition of ionic liquids such as* N*,*N*-diethyl-*N*-methyl-*N*-(2-methoxyethyl)ammonium bis(trifluoromethanesulfonyl)imide (DEME-TFSI) can enhance the ionic conductivity of Mg(HMDS)_2_-MgCl_2_/THF electrolyte [87]. The highly dissociative DEME-TFSI salt was found to form free ions up till 53.2 mol% of DEME-TFSI in 0.5 M Mg(HMDS)_2_-MgCl_2_/THF. This highly dissociative ionic liquid does not affect the first coordination sphere of Mg(HMDS)_2_-MgCl_2_/THF, but disrupts the second solvation shell. A single peak observed in NMR suggested a rapid exchange or dissociation in the solution by disrupting the complex ion pair formed between and [HMDSMgCl_2_]^−^ and forming a weak ion pair between and [TFSI]^−^. Such rapid exchange of ions can possibly result in structural diffusion, leading to enhanced ionic conductivity and current density of the solution. It should be noted that the structure of the cation complex observed here ([{(THF)_3_MgCl}_2_-µ-Cl]^+^) is different than the typical dimer cation complex observed in the previous study for organometallic electrolyte where three chlorine ions are shared by two magnesium ions, whereas here only one chlorine forms the bridge between two magnesium ions.

**Fig. 14:**
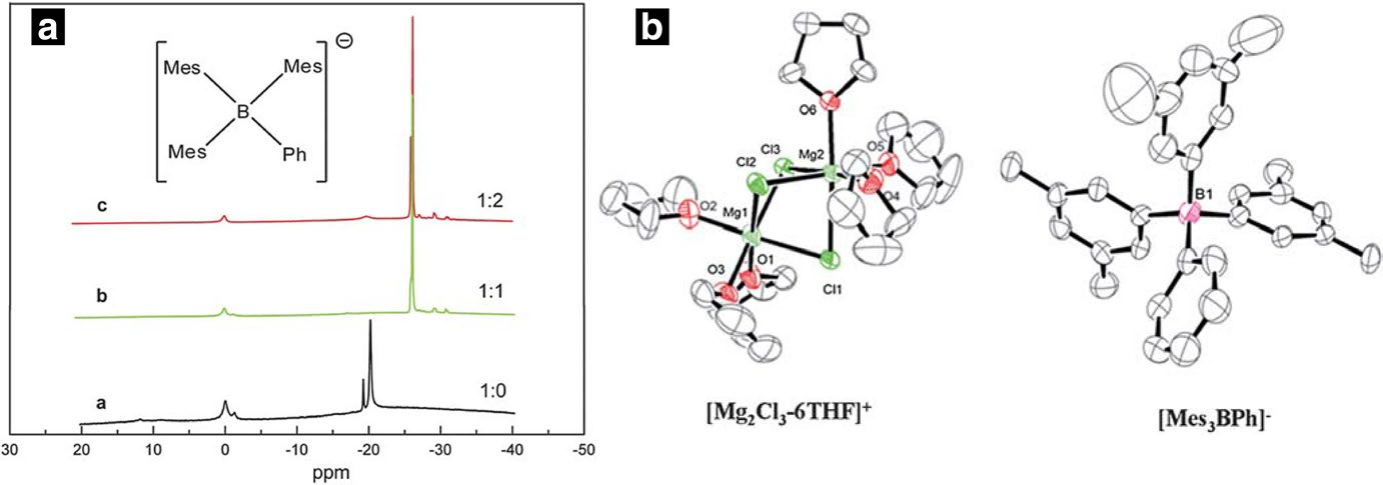
**a** ^11^B NMR spectra measured with boron-based electrolyte solutions of Mes_3_B/THF (**a**), Mes_3_B–PhMgCl/THF(**b**), Mes_3_B–(PhMgCl)_2_/THF (**c**), (**b**) ORTEP drawing of the molecular structure of the crystallized Mes_3_B–(PhMgCl)_2_ complex electrolyte. Hydrogen atoms have been omitted for clarity. Reproduced from Ref .88 with permission from The Royal Society of Chemistry

Organic boron based Mg complex (OMBCs) salts were initially studied by Gregory in 1990 and later by Aurbach in 2002, but they were found to be limited by low cycling efficiency and anodic stability [5, 66]. Recently Guo et al. [88] developed an OMBC through reaction of tri(3,5-dimethylphenyl)borane (Mes_3_B) and PhMgCl in THF. Even though exact intermediates species present in the solution are unclear, based on the single-crystal XRD, NMR, Raman and fluorescence spectra analyses, they reported the same cation complexes (Mg_2_Cl^3+^, MgCl^+^) as observed in DCC, APC and HMDSMgCl electrolytes as the main cationic species and Ph_2_Mg and [Mes_3_BPh]^−^ as anionic species present in the solution. The XRD results suggested the presence of ion paired [Mg_2_Cl_3_-THF_6_]^+^ [Mes_3_BPh]^−^, where the anion is tetrahedrally coordinated, while the cation shows a typical bridged structure of magnesium atoms hexa-coordinated by three chlorine and six THF molecules (Fig. 14). Further, fluorescence spectroscopy for different ratios of Mes_3_B mixed with PhMgCl and Raman spectroscopy analysis suggested the presence of weak interactions between [Mes_3_BPh]^−^ and [Ph_2_Mg] through the formation of aggregates or π–π interactions, which results in high anodic stability of the Mes_3_B-(PhMgCl)_2_ electrolyte solution.

**Fig. 15:**
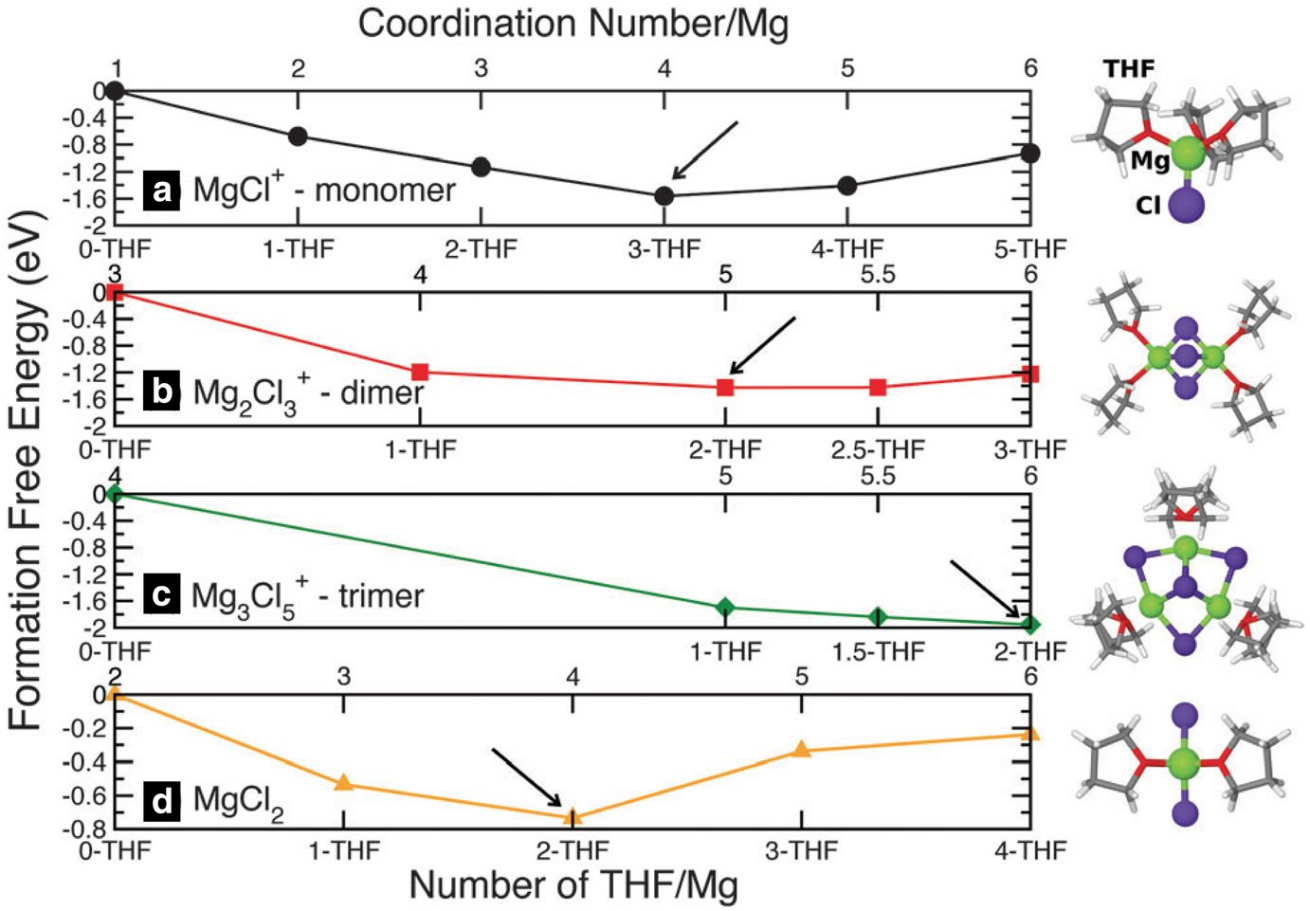
Formation free energy (in eV) of magnesium-chloride complexes as a function of THF coordination for** a** MgCl ^+^ (monomer),** b** Mg_2_Cl_3_^+^(dimer),** c** Mg_3_Cl_5_^+^ (trimer), and** d** MgCl_2_.* Arrows* indicate the most stable THF coordination environment for each complex. Snapshots of the most stable magnesium-chloride complexes are also depicted. Ref. 90 - Published by The Royal Society of Chemistry

Doe et al. reported the first simple all-inorganic Mg electrolyte by in situ reaction between MgCl_2_ and AlCl_3_ in ethereal solutions, namely the magnesium aluminum chloride complex electrolytes (MACC). The MACC electrolyte shows a high anodic stability of ~ 3.3 V with good reversible deposition/dissolution, however, the reported enhanced performance after conditioning remains a mystery and the performance deteriorates after the few cycles [89]. Canepa et al. [90] coupled ab initio calculations with molecular dynamics simulations to investigate the functional species and the structural evolution during electrochemical cycling. In agreement with Wan et al. and Nakayana et al. results for DCC electrolytes, Canepa et al. observed fourfold coordination for monomers (MgCl^+^ and MgCl_2_) and fivefold coordination for dimers (Mg_2_Cl_3_^+^) (Fig. 15). Some experimental studies suggested the formation of trimers and multimeric units in MACC electrolytes [89] and Canepa et al. reported a sixfold coordination of trimer (Mg_3_Cl_5_^+^) complexes [91]. However, Canepa et al. predicted dimers and trimers to be metastable under normal thermodynamic conditions, suggesting that a dimer structure might become accessible with the reduction of the THF chemical potential (e.g., drying). In contrast to these results, other experimental studies using X-ray diffraction, Raman, and NMR spectroscopy revealed hexa-coordinated structure of Mg ions in the MACC electrolytes for both monomer MgCl^+^ as well as dimer Mg_2_Cl_3_^+^ complexes. Four-fold coordination was observed in aluminum complexes, where (AlCl_2_^+^THF_2_), (AlCl_2_^+^ THF_2_), AlCl_3_ (THF) and AlCl_4_^−^ were suggested as the stable species in the solution while no polymeric species were observed. Contrary to experimental studies, the simulation does not report the formation of other higher-order magnesium-chloro structures, rather suggests agglomeration of MgCl^+…^MgCl_2_, which could be interpreted as higher-order clusters in spectroscopic measurements [90, 91]. We note that dimers or other higher-order complexes would be favored under drying conditions due to lack of solvent molecules or solvent polymerization. It was speculated that enhanced electrochemical performance after conditioning is due to increase in the concentration of active species (MgCl^+^ and AlCl_4_^−^) in the solution. Conversely, resting the electrolyte, a process known as ‘aging’, deteriorates the electrochemical performance of MACC electrolytes, which was correlated with the drastic decrease in the concentration of electroactive species in the solution.

### Aqueous Mg Electrolytes

Water is an excellent solvent for the divalent cations considered here, hence aqueous electrolytes are expected to exhibit improved solubility for Mg salts. However, conversely, aqueous electrolytes are limited by the inherent water molecular electrochemical stability window of 1.5 V and passivation of the Mg metal anode. For reasons discussed above, there are sparse studies on aqueous Mg electrolytes for energy storage and those available report mainly simple salts such as Mg(SO_4_)_2_, Mg(ClO_4_)_2_, Mg(NO_3_)_2_ and MgCl_2_. But very recently Seoung-Bum et. al demonstrated reversible cycling of the Mg/V_2_O_5_ full cell in a 0.5 M Mg(TFSI)_2_/PC electrolyte containing 3 M water. They suggested that presence of small amount of water supports the intercalation of Mg ions in V_2_O_5_. Since water is well known to passivate the Mg metal anode, they employed a protective artificial polymeric interphase to suppress the electrochemical decomposition of water-containing carbonate based electrolyte. However, details of the active species present in the electrolyte is not known at this point [92]. Due to the excellent hydration of Mg, exhibiting a preferred octahedral first-shell solvation structure [93], most simple salt aqueous solutions are reported not to exhibit ion pairs below 2 M [94, 95]. Yagi et al. evaluated the Mg(SO_4_)_2_ electrolyte performance and concluded that dehydration plays an important role [96]. As aforementioned, a large desolvation energy is expected in the case of complete desolvation, and partial dehydration at the electrode interface alleviates the penalty of this process. While some hydration may benefit intercalation in oxides [97, 98], it is expected that ions with smaller hydration numbers exhibit less detrimental impact on the host structure [99]. Buchner et al. reveal the dependence of the hydration number on the concentration of Mg(SO_4_)_2_ [100]. It was deduced that the effective hydration number decreases from ~ 14 at infinite dilution to ~ 10 at high concentrations (> 1 M) of Mg(SO_4_)_2_. We note that this effective hydration number includes not only the first solvation shell but also the second solvation shell where the H_2_O molecule that are “irrotationally bounded” [100]. Raman spectroscopy confirmed the formation of both CIPs and AGGs in (> 1 M) Mg(SO_4_)_2_ aqueous solutions [100, 101], which reduces the hydration numbers, in turn affecting the electrolyte electrochemical properties. The association constants of Mg(SO_4_)_2_ were also experimentally determined [40, 102]. However, the direct effect of ion pair formation on electrochemical performance was not investigated.

Several early reports demonstrated that additions of water can facilitate the desolvation process as well as insertion into various vanadium metal oxides as a result of the strong solvation structure between water and Mg^2+^ [102–110]. The highest specific charge, 170 Ah/kg, was attained in a 1 M Mg(ClO_4_)_2_ + 1 M H_2_O solution in acetonitrile, which coincides with a 1:1 ratio between Mg^2+^ and H_2_O [110]. Similarly, Song et al. [111] reported reversible intercalation in a MnO_2_ nanowire electrode with gold current collector and a water-containing Mg(ClO_4_)_2_/PC electrolyte where the highest performance was observed at a ratio of Mg^2+^ to H_2_O ratio of 1:6. However, co-intercalation of water, while alleviating the desolvation process, tends to compromise the structural integrity of the electrode material and hence the cycling stability.

In contrast to non-aqueous solutions, aqueous MgCl_2_ electrolytes have been reported to exhibit weak ion-pairing tendencies (0.2 M Cl^−^, 1.4 Mg^2+^, 3 M ionic strength in a Mg^2+^–Na^+^–Cl^−^–ClO_4_^−^ system) from both potentiometric and osmometric measurements [112]. Furthermore, the association constant of Mg^2+^−Cl^−^ is 25.6 times smaller than Mg(SO_4_)_2_ in aqueous solutions [102], indicating that the Mg^2+^–Cl^−^ ion pair is significantly weaker than for Mg(SO_4_)_2_. For Cl^−^ and SO [42] ^−^, the ion pair stability with divalent metal ions decreases from Ca^2+^ to Mg^2+^, which supports the speculation that the hydration number of Mg^2+^ is larger than that of Ca^2+^ [40].

### Mg Polymer Electrolytes

Polymer electrolytes hold the promise of electrochemical and thermal stability, which are important regardless of the specific chemistry. A primary challenge that remains for practical battery applications of polymer electrolytes is effectively managing the tradeoff between ion transport and other physical or chemical properties, such as mechanical or thermal stability. Solvation structure and specifically ion pairing impacts transport properties and is the focus of this section. Lithium poly(ethylene oxide) (PEO) electrolytes have been well characterized and provide a useful starting point for the discussion of multivalent ions.

Despite PEO having a modest dielectric constant (~ 7.5) [113], it has been reported both experimentally and computationally that CIPs do not appear to form until high salt concentrations, where the number of ether oxygens (EO) per metal cation nearly matches the average coordination number for both Li (5 EO) and Mg (6 EO) with TFSI anion. Mao et al. [114, 115] used neutron diffraction isotopic substitution (NDIS) to identify the solvation structure of high molecular weight, amorphous PEO LiTFSI. It was found that, on average, no CIPs were present in P(EO)_7.5_LiTFSI at room temperature. Although no CIPs were present, broad peaks around 4.85 and 5.5 Å in the pair distribution function were suggested to be the result of SSIPs. Conductivity data provides similar evidence. Conductivity of binary liquid electrolytes generally increases with salt concentration until a maximum is reached where the formation of neutral IPs or neutral ion AGGs lead to a reduction in conductivity [116]. This analysis is complicated for systems where charged IPs or AGGs contribute to conductivity. It was recently demonstrated that charged AGGs (i.e., triplets) are important for transport in highly concentrated PEO LiTFSI [117]. Additionally, in the case of polymers, increased ion concentration reduces segmental motion, further complicating the connection between a maximum in conductivity and the formation of neutral IPs or AGGs. Nevertheless, in the case of high molecular weight PEO (5 and 20 kg/mol) a maximum in conductivity at 363 K and 373 K is reached in the range between P(EO)_15_LiTFSI and P(EO)_10_LiTFSI [118, 119], which at least in part, indicates formation of neutral CIPs or AGGs at higher salt concentrations. Combining NDIS and conductivity data, it appears that SSIPs are present at EO:Li ratios between 10 and 7.5, leading to a reduction in conductivity.

Conventional spectroscopic methods such as IR or Raman, do not typically detect SSIPs, however, these methods can be advantageous for identifying CIPs [4]. IR and Raman studies of high molecular weight amorphous PEO LiTFSI seem to be in agreement that little to no CIPs are present when EO:Li is greater than or equal to 8 [120, 121], and a substantial fraction of CIPs (~ 24%) are present when EO:Li is equal to 6 [121]. It is worth noting that Edman saw little evidence of CIPs in room-temperature samples that were not preheated to the amorphous regime at the same salt concentration [121]. This was attributed to slow recrystallization of salt-rich PEO, and is evidence that crystalline regions solvate Li salts at higher concentrations than amorphous regions.

**Fig. 16:**
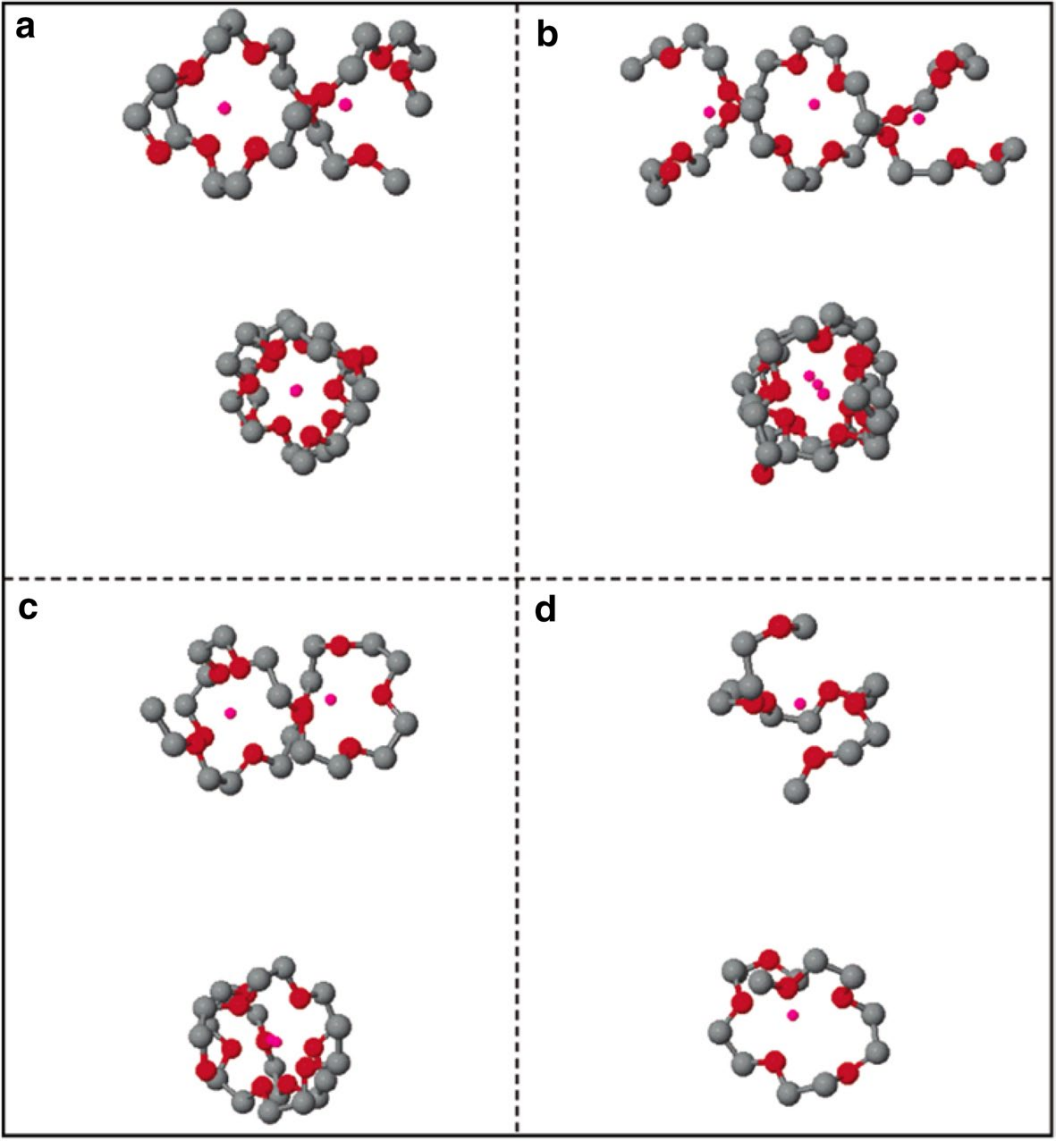
Snapshots from simulations of PEO/LiTFSI at 423 K (**a**,** b**,** d**) for EO:Li = 20:1 and** c** for EO:Li = 10:1. Hydrogen atoms are not shown for clarity. Only atoms within 4.0 Å of each Li + are shown. Reprinted with permission from Ref. 112. Copyright 2006 American Chemical Society

A molecular dynamics study of P(EO)_7.5_LiTFSI at 393 K reported 4.6 oxygen atoms in the first coordination shell, 3.85 from EO, and 0.5 from TFSI anion (it is unclear why these do not sum to 4.6) [122]. The elevated presence of CIPs compared to experiment at this salt concentration may be due to the challenge of obtaining accurate electrostatic interactions including polarization for concentrated solutions. A few simulation snapshots are shown in Fig. 16 to help visualize the local environment around the cations in the PEO matrix. Overall, the picture that emerges for high molecular weight amorphous P(EO)_n_ LiTFSI is that SSIPs are favorable between *n* = 15 and *n* = 10 and CIPs begin to be favorable when *n* < 7.5; CIPs make up a substantial fraction when *n* = 6.

While there is a smaller body of work exploring PEO with Mg salts, several systems are summarized in Table 1. Bakker et al. [115] performed conductivity and FTIR experiments on a variety of divalent cations, including Mg, Ca, Sr, and Ba with the TFSI anion. Conductivity data does not provide unambiguous results with respect to IP formation as previously mentioned. However, FTIR indicates that CIPs are not favored until *n* < 9 in P(EO)_*n*_MTFSI_2_, which is, as excepted, higher than that of the lithium analog due to magnesium’s larger average coordination number. Additionally, a larger shift in the C–O–C vibration band was observed for Mg relative to Ca, Sr, and Ba, demonstrating that smaller cations interact more strongly with ether oxygens [115]. In contrast with Edman’s LiTFSI study, the authors found no CIP temperature dependence in the range tested. This can be reconciled by the absence of crystalline peaks in multivalent systems at concentrations *n* ≤ 16. Specifically for multivalent applications, it is interesting to note that the strong interaction between Mg^2+^ and ether oxygen atoms distorts crystalline regions. These observations were supported by Edman, as well as by Reddy and Chu who observed reduced PEO crystalline content with increasing salt concentration in a PEO Mg(ClO_4_)_2_. FTIR and conductivity measurements were combined to detect CIPs, and for P(EO)_*n*_Mg(ClO_4_)_2_ CIPs were observed as favorable when *n* < 28 [123]. We note that although this value is considerably larger than that found by Bakker et al., it is likely due to the different anions present.

**Table 1:** Comparison of the CIP regime, the point at which there are more CIPs than SSIPs and free ions, for a variety of electrolyte systems and the methods that aided in this determination

Electrolyte system	CIPs regime	Methods
P(EO)_n_LiTFSI	*n* ≤ 7.5	NDIS [114], FTIR [120, 123], Ramen [120], Conductivity [118, 119]
P(EO)_n_Mg(TFSI)_2_	*n* < 9	FTIR, Conductivity [115]
P(EO)_n_Mg(ClO_4_)_2_	*n* < 28	FTIR, Conductivity [123]
P(EO)_n_δ-MgCl_2_	*n* ≤ 32	FTIR [124, 166] Conductivity [124]
PEO-b-P[(STFSI)_2_Mg]	Not Dissociated	SAXS, Conductivity [127]
PEO/B/PEO Mg(ClO_4_)_2_	N/A	Ramen, Conductivity [128]
(MEEP)_n_ Mg(TFSI)_2_	*n* < 8	Conductivity [129]

In a short-chain P(EO)_*n*_δ-MgCl_2_ (referred to as poly(ethylene glycol) PEG) system it was formulated, using FTIR, that free ions dominate when *n* ≥ 86, and both Mg^2+^ and MgCl^+^ are present when 14 ≤ *n* ≥ 32 [124]. Both Mg^2+^ and MgCl^+^ contribute to conductivity and using equivalent conductivity analysis a strong cation–anion interaction was demonstrated with increasing concentration [125].  Additionally, it is interesting to note that Di Noto el al. observed increased IP, either MgCl^+^ or MgCl_2_, for given O:Mg ratios when the short PEO chains were linked together using diethoxydimethylsilane (DEOS) PEO/DEOS/PEOδ­-MgCl_2 _[126].

Single–ion conductors or ionomers utilizing a PEO type domain have recently gained attention as the concept introduces the possibility to have a cation transference number near unity by covalently bonding the anion to the polymer backbone [130]. A recent study tested both Li and Mg single-ion block copolymers, PEO-b-P[(STFSI)Li] and PEO-b-P[(STFSI)_2_Mg] [127]. It was found that the conductivity of the magnesiated samples were about an order of magnitude lower than that of the lithiated samples. This was attributed to roughly an order of magnitude lower number of charge carriers in the Mg system when fitted with the Vogel–Tamman–Fulcher (VTF) equation. Additionally, there was a notable difference in the small-angle X-ray scattering (SAXS) profile. The SAXS peak present in magnesiated samples revealed local correlations between like blocks of different chains. The hypothesis put forward was that Mg^2+^ did not dissociate from the P[(STFSI)_2_Mg block, remaining a CIP, causing less mixing than in the Li-containing system.

Lewis acidic polymer electrolytes or introducing Lewis acid moieties have also been investigated [131]. In PEO, strong cation–polymer interactions aid in the dissociation of ions, however as a result, conductivity is dominated by anion motion [132]. On the other hand, Lewis acid groups can interact strongly with anions, promoting cation motion and dissociation while inhibiting anion motion. Saito et al. cross-linked short PEG chains with boron ester (Lewis acid) groups, and tested the cross-linked system with a variety of Mg salts. As expected, cation transference increased with increasing boron ester concentration. Interestingly, the effect was anion-dependent, ClO_4_^−^ being more easily immobilized than TFSI^−^. At both EO:Mg ratios, 64 and 32, the relative proportion of AGGs and CIPs decrease while ion dissociation increased (shown by Raman) as compared to a system with a lower concentration of borate ester group [128]. Unfortunately, the conductivity also dropped by orders of magnitude with the increased boron ester group concentration, indicating that the cation transference number increased due to immobilized anions rather than improved cation mobility in PEO-type electrolytes.

A different type of polymer that has been investigated for Mg electrolytes is poly(bis(2-(2-methoxyethoxy)ethoxy)phosphazene) (MEEP) [129], which has oligoether side groups similar to PEO. The behavior of both Li and Mg salts were investigated in this polymer. Impedance measurements showed that MgTFSI_2_ in MEEP exhibited roughly the same maximum conductivity as LiTFSI, although the maximum was reached at different concentrations MEEP: Mg = 8 and MEEP: Li = 4, respectively. In both cases, it was suggested that anion movement accounted for most of the conductivity, and the concentration difference was rationalized by the Mg salt having two TFSI^-^ anions. MEEP MgTf_2_ exhibited low conductivity even at low concentrations (MEEP: Mg ≥ 20), and the authors postulated that this was due to extensive ion association.

In all of the PEO Mg examples thus far, with the exception of Reddy and Chu and Saito et al., conductivity data was interpreted with the VTF equation. Remarkably, all the data was reasonably well fit by the VTF Eq. 1;

1 $$\sigma (T) = \frac{A}{\sqrt T }e^{{ - {B \mathord{\left/ {\vphantom {B {R\left[ {T - \left( {T_{g} - 50} \right)} \right]}}} \right. \kern-0pt} {R\left[ {T - \left( {T_{g} - 50} \right)} \right]}}}}$$

where *A* and *B* are generally interpreted as the number of charge carriers and pseudo-activation barrier respectively [127]. This physical interpretation of the equation is obviously overly simplified even for Li [133], let alone multivalent ions where multiple types of charge carriers exist. For example, the activation barriers for a monovalent CIP, Mg^+^ with an anion, as compared to a free Mg^2+^ ion are expected to be quite different. Hence, conceptualizing similar physically motivated equations that take into account multiple types of charge carries would be of interest to the community.

As previously mentioned, a major appeal of polymer electrolytes is electrochemical stability. PEO has been shown to have good stability in Li systems [134], but little work has been done to demonstrate stability with Mg or other chemistries. Most studies to date have not cycled Mg polymer electrolytes in a full cell configuration. There is one report of PEO/MgO Mg(BH_4_)_2_ where the system (Mg| PEO/MgO Mg(BH_4_)_2_ | Mo_6_S_8_) indicated good stability over 150 cycles [51]. Although a detailed investigation of the solvation structure for Mg(BH_4_)_2_ in PEO was not conducted, it was demonstrated that increasing glyme (glymes are PEO oligomers) length decreased the strong Mg BH_4_ interaction resulting in increased ion dissociation. The authors extrapolated that greater dissociation could be expected in PEO [51]. One aspect of stability that has not been explored is the stability of polymer solvents in the presence of partially reduced Mg^+^, which is a likely transient specie during Mg plating and has been shown to be highly reactive causing anion or solvent decomposition [30].

Another difference to highlight between Li and less dendrite-prone multivalent metals such as Mg is that the polymer electrolytes may not need to provide the same amount of mechanical support to prevent dendritic growth. This allows for the use of plasticizers and inclusion of organic [135, 136] or ionic liquids [137, 138] to make polymer gel electrolytes or polyelectrolytes. An in-depth study of IP in polymer gel electrolytes or polyelectrolytes is outside the scope of this paper, however, it would be expected that this addition would increase the dielectric constant of the solvent thus reducing IP and potentially increasing the conductivity of the system. On the other hand, if a volatile or electrochemically less stable compound is added, it may negate the inherent safety benefits of polymer electrolytes and hence tradeoff effects are to be expected.

## Zinc Electrolytes

Zinc has been implemented in various commercial primary battery systems, in particular, the famous “alkaline” battery with a MnO_2_ cathode and aqueous alkaline electrolyte. However, in the early development of secondary zinc batteries, poor cycling performance was observed for this cell configuration due to undesirable zinc morphologies such as dendrite growth, as well as zinc redistribution (shape change) on cycling, both of which are linked to the high solubility of zinc in alkaline media [7, 139]. In addition, there are safety and environmental issues associated with the corrosive nature of an alkaline electrolyte. The introduction of near-neutral pH electrolytes with salts such as ZnSO_4_ and Zn(NO_3_)_2_ greatly curbed cycling issues related to zinc dendrite formation and enabled nearly full capacity retention up to 100 cycles [10]. Further improvements to cycle life stemmed from cathode material development, recently leading to reports such as a vanadium bronze, Zn_0.25_V_2_O_5_·nH_2_O cathode demonstrating up to 80% retention over 1000 cycles [11]. Another recent report with a conversion-based MnO_2_ nanofiber cathode has shown a capacity retention of 92% over 5000 cycles [140]. These cell configurations, utilizing neutral aqueous electrolytes, benefit from advantages associated with water such as high conductivity and low cost; in addition, water does not suffer from the volatility and flammability of some organic solvents in non-aqueous electrolytes. The solvation structure of Zn aqueous electrolytes could be expected to contain little ionic association due to the excellent solvation ability of water, similar to that of Mg aqueous electrolytes. Raman spectroscopy of the SO_4_^2−^ anion, commonly used in aqueous Zn electrolytes, shows shifting of the *v*_1_(*A*_1_) symmetric stretch band to higher wavenumber upon contact with Zn^2+^ [141]. From fitted band intensities, the population of ZnSO_4_ as ion pairs can be estimated as 6 ± 2% at 0.389 M to 13 ± % at 1.945 M at room temperature [141]. ZnSO_4_ is typically used in aqueous electrolytes for secondary batteries at concentrations of 2 M or less.

Secondary Zn cells with non-aqueous electrolytes enable larger operating cell voltages (limited to 1.2–1.5 V in aqueous electrolytes, outside of which water hydrolysis and electrolyte loss occurs) as well as greater operating temperatures due to wider liquid temperature ranges. At present, research into non-aqueous secondary zinc energy storage is nascent and overshadowed by analogous Mg versions due to factors such as the relatively high reduction potential of Zn (− 0.76 vs. − 2.4 V of Mg). Nonetheless, zinc retains many benefits in prospective secondary energy storage such as safety, geological abundance, and cost, in addition to a large volumetric capacity (5851 vs. 3833 mA h/ml of Mg), and its development in non-aqueous systems is a compelling avenue of research, especially for grid applications. A recent study by Rajput et al. examining the solvation structure and zinc compatibility of Zn(TFSI)_2_, Zn(CF_3_SO_3_)_2_, Zn(BF_4_)_2_, and Zn(PF_6_)_2_ salts in diglyme (G2), acetonitrile (AN), propylene carbonate (PC), and* N*,*N*-dimethylformamide (DMF) solvents (0.1 M and 0.5 M concentration) demonstrated reversible plating/stripping for the AN − Zn(TFSI)_2_, AN − Zn(CF_3_SO_3_)_2_, and PC − Zn(TFSI)_2_ electrolytes, with anodic stabilities up to 3.7, 3.5, and 3.4 V vs. Zn/Zn^2+^, respectively [12]. This broad assessment of non-aqueous Zn electrolytes with several common anions and organic solvents shows the promise of those containing the TFSI^−^ and CF_3_SO_3_^−^ anions in particular.

As a viable candidate for MV electrolytes, TFSI^−^ has been studied extensively in terms of the anion’s structural flexibility and manner of cation–anion binding in different electrolytes. In the free TFSI^−^ anion, the *trans* conformer is more stable than the *cis* conformer, which can be explained by relief of repulsive steric interactions between the CF_3_ groups present in the *cis* conformer. Early theoretical work estimated the enthalpy difference between the two conformers as 3.5 kJ/mol at the B3LYP/6-311 + G(3df) level of theory [142]. Subsequently, an experimental report put the enthalpy difference at 2.2 kJ/mol using IR spectroscopy [143] and another in the 3.4–7.3 kJ/mol range using Raman spectroscopy [144]. Accordingly, the conformational equilibrium of non-coordinated TFSI^−^ (e.g., in ionic liquids) generally shows preference for the *trans* conformer [144]. However, the *cis* conformation becomes favorable in the coordination sphere of metal cations [145], where an increase in binding strength is associated with an increase in the dipole moment of the anion from 0.301 D in the *trans* conformer to 4.382 D in the *cis* conformer [142]. Other studies detailing the structural nature of Zn complexes with TFSI^−^ have shown a preference for the *cis*-conformer of TFSI^−^ anions in the coordination sphere using UV–Vis, IR, and Raman spectra [146]. In addition, a recent computational study of [Emim][Zn(TFSI)_3_] (Emim = 1-ethyl-3-methylimidazolium) complexes at the B3LYP/6-31G(d) level of theory suggested that zinc forms a homoleptic octahedral complex with all the TFSI^−^ anions bound to Zn in the *cis* conformation [147]. The Mg^2+^ cation, of similar ionic radius, shows similar coordination behavior with TFSI^−^ in ionic liquids, though some solvation studies which include IR/Raman spectroscopy have observed frequencies associated with bridging TFSI^−^ anions [32]. Similar aggregate formation could easily be expected from analogous Zn systems.

In non-aqueous electrolytes consisting of the TFSI^−^ salt in some organic solvent, weakly coordinating behavior of the TFSI^−^ anion could rationally be expected due to the flexible nature of the anion and its delocalized charge. However, theoretical calculations such as MD simulations have often shown this not to be the case; ion–ion association of TFSI^−^ with Mg or Zn is prevalent in solutions with common organic solvents even in modest concentrations (0.4 M–0.5 M) [12, 30]. Though this ionic association increases the oxidative stability of the anion [30], the formation of multi-meric species negatively affects the dynamics of the electrolyte in terms of conductivity, viscosity, and diffusion, especially if there is a tendency toward higher-order aggregate formation. In addition, as noted in Sect. 1.1, ion–ion association between Mg^2+^ and TFSI^−^ is known to render the anion susceptible to decomposition upon reduction of Mg^2+^ to Mg^1+^, which may play a role in the anode passivation observed at concentrations at or above 0.5 M for electrolytes with this salt [30]. The extent of ion–ion association and aggregate formation can be predicted in part by first-principles calculations of cation–anion binding strength. A computational study comparing M(TFSI)_2_ complexes across several metals (M = Mg, Ca, Ba, Zn, and Cu) showed that the binding energy between the metal and the TFSI^−^ ligands is stronger for Zn^2+^ than Mg^2+^ by 43.2 kcal/mole at the B3LYP/6-31G*//B3LYP/lanl2dz level of theory [148]. Thus, one may expect greater tendency for ion–ion association and decomposition of TFSI^−^ in non-aqueous Zn electrolytes than the corresponding Mg ones. However, to our knowledge, no electrode passivation has been reported with an organic Zn(TFSI)_2_ electrolyte, even at 0.5 M concentration. A closer inspection of the solvation structure and stability against decomposition is desirable to probe any relationship between TFSI^−^ stability and the ion paired cation (Zn or Mg).

**Fig. 17:**
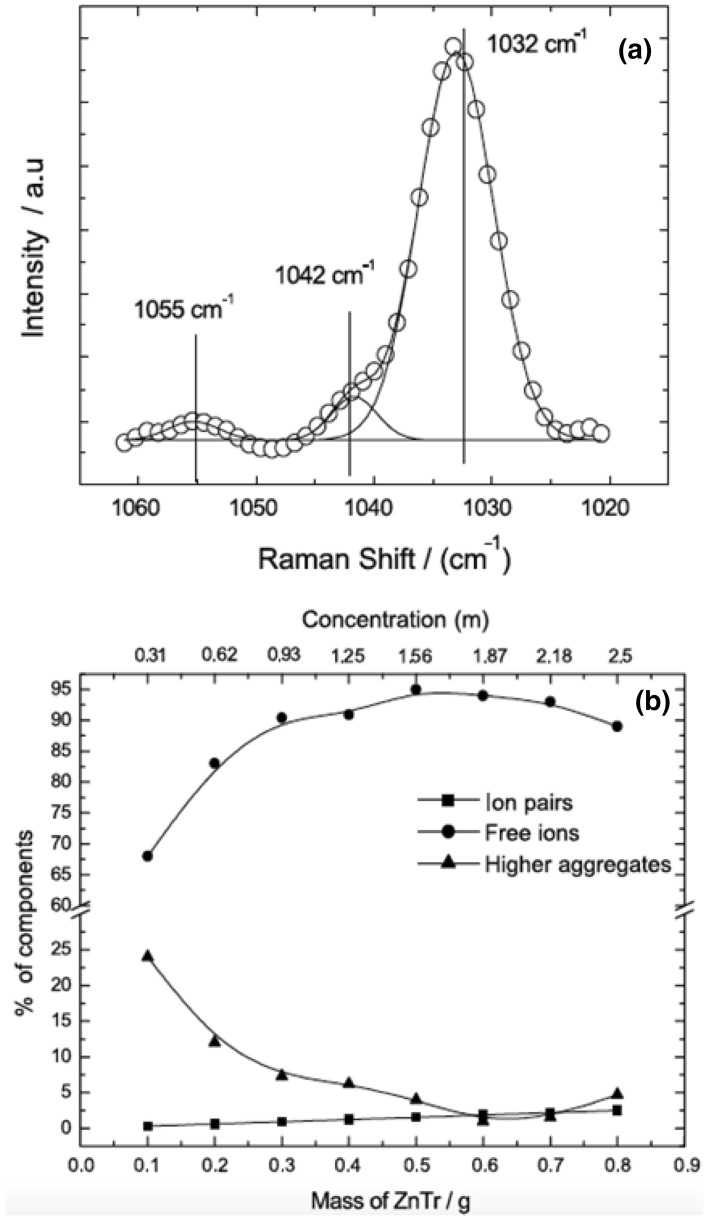
**a** Spectrum of the symmetric SO_3_ stretching region of an electrolyte of polyacrylonitrile (0.25 g), propylene carbonate (0.6 g), ethylene carbonate (0.6 g), and Zn(CF_3_SO_3_)_2_ (1.25 M). The band at 1032 cm^−1^ corresponds to free CF_3_SO_3_^−^, 1042 cm^−1^ to ion pairs and 1055 cm^−1^ to higher aggregates.** b** Percent composition of free ions, ion pairs, and higher aggregates as a function of salt concentration. ZnTr = Zn(CF_3_SO_3_)_2_. Reprinted from Ref. 148, with permission from Elsevier

Zinc gel polymer electrolytes have also received considerable attention. Zn(CF_3_SO_3_)_2_ is a commonly employed salt in gel polymer electrolytes, perhaps to follow implementation of the CF_3_SO_3_^−^ anion in lithium electrolytes. In addition to its high anodic stability as noted above, the CF_3_SO_3_^−^ anion is known for its chemical stability and “weakly coordinating” nature, potentially making it another good candidate for MV electrolytes. Raman spectroscopy of the CF_3_SO_3_^−^ anion shows splitting of the symmetric SO_3_ stretching mode upon coordination to a metal cation [149]. Bandshape analyses of spectra in conjunction with conductivity measurements can show the extent of ion–ion association in different salt concentrations of an electrolyte, with higher conductivity typically associated with higher populations of “free” CF_3_SO_3_^−^ ions [149–151], e.g., Fig. 17 shows the Raman spectrum and percent composition of a Zn(CF_3_SO_3_)_2_ gel polymer electrolyte as free ions, ion pairs, or higher aggregates at different salt concentrations. In other polymer-based electrolytes, containing e.g., PEO, ion pairing is qualitatively known to be pervasive for many salts from spectroscopic methods such as EXAFS [152, 153].

**Fig. 18:**
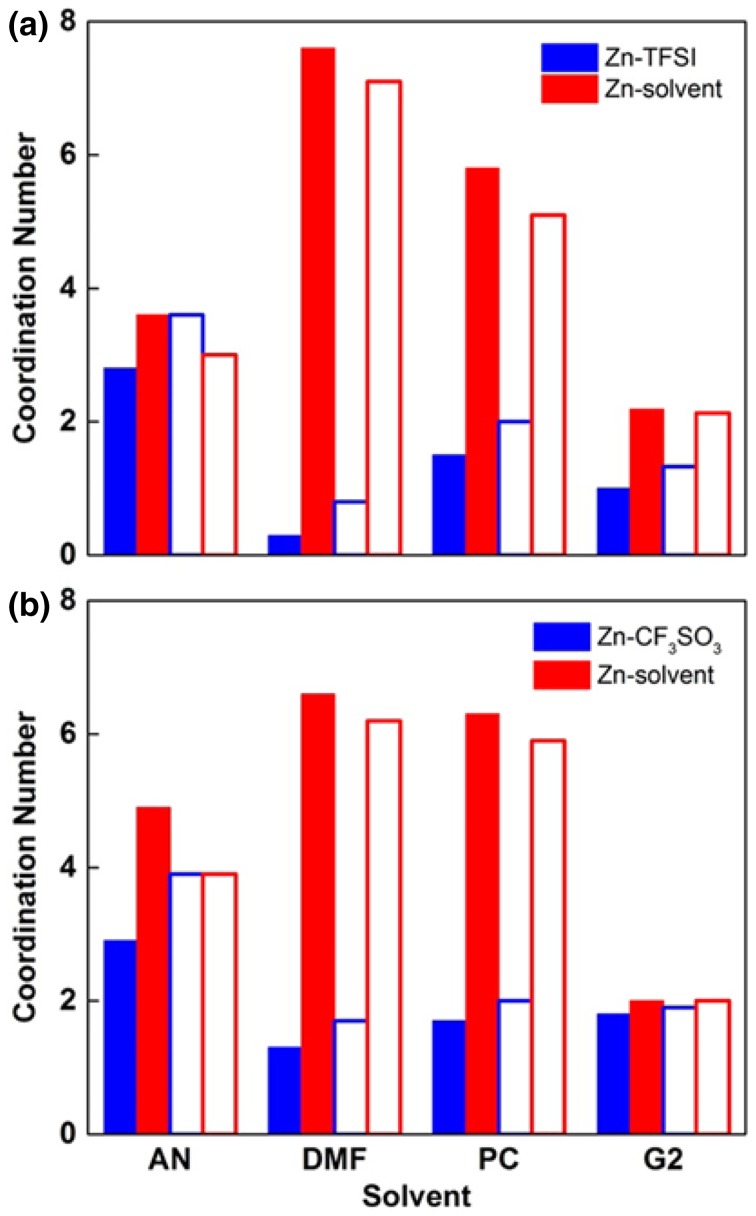
Coordination numbers of Zn-anion and Zn-solvent for TFSI^−^ (**a**) and CF_3_SO_3_^−^ (**b**) at 0.1 M (*filled bars*) and 0.5 M (*hollow bars*) for four different solvents. Reprinted with permission from Ref 12. Copyright 2016 American Chemical Society

The neutral Zn(CF_3_SO_3_)_2_ complex exhibits four oxygen atoms, two from each CF_3_SO_3_^−^ ion, bound tetrahedrally to the Zn^2+^ center [148]. In non-aqueous solutions, the coordination behavior is more complicated and also involves molecules from the solvent, which compete with or join alongside the anion(s) for Zn^2+^ coordination. In general, the coordination number for Zn^2+^ in non-aqueous Zn(CF_3_SO_3_)_2_ solutions exceeds four and ion–ion association is significant at 0.1 M and 0.5 M concentrations across a range of organic solvents [154]. It may be of interest to note that the binding energy between Zn and the anions in Zn(CF_3_SO_3_)_2_ is stronger than for Zn(TFSI)_2_ by 40.8 kJ/mol at the B3LYP/6-31G*//B3LYP/lanl2dz level of theory [148] with possible ramifications on the relative extent of ionic association in non-aqueous solutions. A direct comparison of the solvation structure between Zn(TFSI)_2_ and Zn(CF_3_SO_3_)_2_ electrolytes using MD simulations [12] corroborates this finding (see Fig. 18). Of the solvents examined at 0.1 M and 0.5 M, in the weakly coordinating solvents acetonitrile and propylene carbonate, the coordination number of Zn from TFSI^−^ ligands is similar to that of CF_3_SO_3_^−^, whereas in the strongly coordinating solvents* N*,*N*-dimethylformamide and diglyme, the coordination number from TFSI^−^ is slightly smaller. The coordination number in both Zn-TFSI^−^ and Zn-CF_3_SO_3_^−^ increases from 0.1 M to 0.5 M, but more so for TFSI^−^, due to stronger solvent-anion interactions in CF_3_SO_3_^−^.

## Calcium Electrolytes

Among the multivalent ions, calcium faces some of the most serious hurdles in the development for practical electrochemical secondary energy storage. However, Ca may be gaining momentum in light of some recent exciting reports. Ca is an appealing component for high-voltage batteries due to its low standard reduction potential (− 2.87 vs. − 2.38 V for Mg, and almost as low as Li at − 3.04 V). Other advantages of Ca are those shared with other multivalent metals such as geological abundance, safety, lack of toxicity, and cost.

Early assessment of nonaqueous Ca electrolytes with salts such as Ca(ClO_4_)_2_ and Ca(BF_4_)_2_ in some common organic solvents exposed them as incompatible with calcium metal due to formation of a oxide passivation surface layer with prohibitively low Ca transport [6]. Due to some success of Grignard reagents in early secondary Mg battery research, it was reasonable to explore the analogous Ca salts, the so-called heavy Grignards (RCaX, where X is a halide). However, no reports have been made of reversible plating/stripping of calcium metal with an electrolyte containing these salts. We also note that the synthesis of heavy Grignards can face difficulties due to the reduced reactivity of Ca metal compared to Mg [155].

Hence, until recently, secondary Ca battery development was impeded by the lack of electrolytes, while research on secondary Mg batteries has flourished in comparison. Finally, a report by Ponrouch et al. showed plating/stripping of calcium metal with electrolytes containing Ca(ClO_4_)_2_ and Ca(BF_4_)_2_ in ethylene carbonate and propylene carbonate. Successful plating/stripping took place despite formation of an anode surface layer, which was formed through electrolyte decomposition as confirmed through chemical analysis of anode surface deposits, and elevated temperatures were required to enable ionic transfer through the anode surface layer; still exhibiting with a ~ 2 V overpotential [139].

Assembly of a full working secondary cell requires a suitable cathode materials that enable facile intercalation/deintercalation of Ca^2+^ ions [156]. Some promising cathodes were developed for aqueous [157] and high water content nonaqueous (17%) [158] electrolytes where successful intercalation/deintercalation was believed to be the result of hydrated Ca^2+^ ions, which reduce the electrostatic interaction between the mobile ion and the lattice of the cathode. Finally, a fully working secondary calcium battery, albeit with low voltage and capacity, was demonstrated for the first time, based on a manganese hexacyanoferrate cathode, a tin anode, and a nonaqueous electrolyte containing Ca(PF_6_)_2_ [159].

Concurrent with the limited research on Ca electrolytes, information on the solvation structure relevant to Ca battery applications has received little attention in the literature. A few general comments can be made based on the available studies and fundamental characteristics of coordination behavior and ion pairing with anions commonly implemented in multivalent ion electrolytes. A Ca^2+^ ion exhibits a larger radius (0.99 Å) as compared to Mg^2+^ (0.65 Å) [160], which results in higher preferred coordination numbers in solution with longer metal to donor atom interaction distances. The binding energy between the metal and anions is less for Ca than Mg in M(TFSI)_2_ by 95.1 kcal/mol at the B3LYP/6-31G*//B3LYP/lanl2dz level of theory [148]. One explanation for the dissimilar binding strength is a different mismatch between the ‘hardness’ of the cation and anion, where the greater hardness of Mg^2+^ is a better match with the hard base character of the TFSI oxygen atoms. Ca^2+^ is less polarizing than Mg^2+^, resulting in a cation–anion interaction with less covalent character.

In summary, while examination of solvation structure in Mg and Zn electrolytes have received considerable attention, Ca electrolytes are less explored. The relationship between the coordination number and binding strength and properties such as conductivity, viscosity, and diffusion for Ca electrolytes is a topic that should be explored further in the coming years of exploratory secondary multivalent ion battery research.

## Rational Design of Electrolytes

A requirement of secondary multivalent-ion battery electrolytes is compatibility with the metal anode, but properties such as high dissociation of the salt and a wide electrochemical stability window are also desirable. These properties, and their dependence on electrolyte speciation, can today exhaustively explored using computations. Depending on computational assets as well as suitable software infrastructure [161], some of these considerations can rapidly evaluated in silico prior to experimental evaluation as part of well-guided electrolyte development. For example, any chemical instability of electrolyte species affecting reversible plating/stripping can be evaluated by first-principles calculations of possible decomposition mechanisms, in particular involving partially reduced, transient radical monocation species. Following the discovery of the decomposition mechanism for the TFSI^−^ anion in the presence of Mg^+^, stability against this and other decomposition mechanisms such as hydrolysis were criteria in the computational design of improved electrolyte anions by Qu et al. involving modifications of TFSI^−^ [162]. The improved anions were predicted by MD simulations to engage in ion association about as much as the parent TFSI^−^ anion. Therefore, the primary method of rational design of electrolytes achieved in this case is enhanced chemical stability in conditions of solvation structure where ion association is prevalent.

Ideal electrolyte anions are non- or weakly coordinating. The chemical stability of anions exposed to transient Mg^+^ would be theoretically less concerning the weaker the ion association. Ion-association strength can be predicted from first-principles calculations, bearing in mind competing ion–solvent interactions. In the absence of ion–ion interactions, the requirement for stability against Mg^+^-driven decomposition should instead fall on the solvent; however, detailed examination of the stability of solvents in this situation by e.g., theoretical calculations is as yet unexplored. One criteria for ion coordination strength is the size of the anion, with bulkier anions expected to be less weakly coordinating, especially if the charge is delocalized, however at the expense of increased viscosity and decreased conductivity of the electrolyte. On the other hand, computed ion-association strength and anion radius only exhibit strong correlation for anions up to a relatively small size [163]. Altogether, small, weakly coordinating, and chemically inert anions are ideal for multivalent battery electrolytes. The recently reported monocarborane anion [61] meets these criteria and is a significant step in the “right” direction. One could imagine that this anion could serve as a platform for further improvements. Indeed, substitution of the hydrogen atoms of this anion with halogens can raise the anodic stability quite significantly compared to the parent anion [164]. However, investigation of the practical implementation of these anions derivations in energy storage applications is premature. In general, halogenation is a common and reliable strategy for raising anodic stability of anions [163]. However, the use of chlorine may be cautioned against, as chloride ions are known to cause corrosion in stainless-steel current collectors [165].

The anodic stability of the electrolyte can be significantly affected by the solvation structure. For example, ion association raises the anodic stability of an anion as compared to the anion by itself. Similarly, cation–solvent interactions raise the anodic stability of solvent compared to the solvent alone. Conversely, anion–solvent interactions decrease the anodic stability of the solvent [166]. The electrochemical stability of an electrolyte is the result of a complex interplay of interactions in which the component having the lowest stability sets the overall limit. In further exploring improved electrolytes for multivalent ion energy storage, the current emphasis on salts of ever-increasing chemical stability, anodic stability, and dissociative behavior will inevitably call for solvent improvements. In “state-of-the-art” electrolytes such as Mg[(CB_11_H_12_)_2_]/tetraglyme, the anodic limit is set by the solvent [61]. Similarly, in the recently reported Mg[B(hfip)_4_]_2_/DME electrolyte with excellent coulombic efficiency (> 98%) and conductivity (6.8 mS cm^−1^) the anodic stability (4.3 V vs. Mg) is limited by the solvent [167]. Therefore, development of improved multivalent battery electrolytes may benefit from a concurrent focus towards the solvent component of the electrolyte.

## Conclusions

The high charge density of multivalent ions typically results in greater tendency to form ion pairs and aggregates in most simple inorganic salts and complexes in organometallic solutions. For example, for simple inorganic Mg and Zn salts, a strong tendency of ion pairing has been observed in most solvents at moderate concentrations, however some high dielectric solvents such as DMSO and some low dielectric solvents with high oxygen donor denticity such as glymes result in solvent separated ion pairs at similar concentrations. The interaction energy between cation and anion tends to be slightly stronger for Zn as compared to Mg ion pairs, with possible ramifications on the relative extent of ion pairing and corresponding effects on conductivity and viscosity of the electrolyte. While MD simulations show significant ion pairing at 0.4 M and 0.5 M in many nonaqueous electrolytes for both Mg and Zn, it may be beneficial to compare the solvation structure more closely in terms of the differences in interaction energies for coordination complexes formed in these electrolytes. Anions with bulky structure and more dispersed charge such as TFSI^−^ show lesser tendency towards ion pairing compared to smaller anions such as BF_4_^−^ and BH_4_^−^ for both Mg and Zn. However, TFSI^−^ was found to exhibit a structural instability during Mg plating, hypothesized and computationally verified as a result of contact ion-pair exposure to the transient Mg^+^ specie. Curiously, this instability has not been reported while ion paired with Zn^+^. In contrast, and in agreement with experimental results, BF_4_^−^ and BH_4_^−^ remain stable during the same charge transfer process. Concentration also plays an important role in determining the structure of ionic species. More mono-dentate configurations of TFSI^−^ anions were observed at lower salt concentrations, and increased bi-dentate configurations at higher concentrations. This development not only leads to an increase in ion pairs and aggregates, but also a decrease in conductivity and an increase in the viscosity of the solution. For magnesium organometallic electrolytes, it is rare to observe naked Mg^2+^ ions, indicating different complex species present in the solution. Most organometallic electrolytes propose a six-coordinated cation aggregate, [Mg_2_(μ-Cl)_3_THF_6_]^+^ as the active Mg^2+^ species and the electrochemical performance and stability varies with the choice of anion which often exists as part of an anionic aggregate in the solution. However, a few studies also reported four- and five-coordination for Mg^2+^ in the aggregates, but in all cases two coordinated Mg^2+^ ions are bridged by three chlorine atoms and form a complex ion pair with the counter anion aggregate/complex. Contradictory results in the literature regarding the coordination number of Mg may be due to inherent approximations in simulation methods, ex situ experimental results and interpretation. Classical molecular dynamics simulations have been widely used to study the solvation structure of ions in solution; however, the accuracy of the predicted coordination environment strongly depends on the force field parameters. For example, a +2 charge on cation in the case of Mg^2+^ and − 1 charge on anion results in hexacoordination of Mg ions, while reduced charges for cations and anions obtained from ab initio calculations results in tetra-coordinated Mg ions [90]. Accordingly, monomers and dimers are observed in reduced charges and whole charges respectively. For aqueous Mg electrolytes, most simple salts do not favor ion pair formation below 2 M, although ion-pairing in Mg(SO_4_)_2_ and MgCl_2_ have been reported. Ion-pairing reduces the hydration numbers, which in turn affects the electrolyte electrochemical properties as aqueous systems often operate by co-intercalation of water. While smaller hydration numbers exhibit less detrimental impact on the electrode materials, it was demonstrated that some hydration may benefit intercalation in oxides. The solvation environment of Mg salts in PEO-type polymer electrolytes is not only concentration dependent, but greatly influenced by the anion present, due to varying strengths of attraction between the cation and anion. When a MgTFSI_2_ PEO system was compared to the analogous Li system, Mg was found to be more highly coordinated by oxygen, which is consistent with the higher charge density. Although PEO-type solvents are capable of solvating and at least partially dissociating multivalent salts, the conductivity remains low due to the strong ether oxygen cation attraction which limits polymer segmental motion. Similar to Li systems, anions are hypothesized to carry the majority of the current. As mentioned, classical molecular dynamics simulations and other ab initio calculations help develop a more comprehensive picture of solvation structure accessing spatial and temporal scales difficult to attain purely through experiment. To the best of our knowledge, there are no such studies on Mg PEO systems. Calcium secondary batteries are relatively underdeveloped, and as a result, the solvation structure of Ca nonaqueous electrolytes is relatively unexplored and should be further investigated. Nuclear magnetic resonance (NMR) has been used widely to study the solvation structure of electrolytes; however, it is limited by the temporal scale, which makes it difficult to de-convolute the peaks and assign them to specific speciation present in the solution using experiments alone, especially in novel electrolyte systems. Also, any change in the charge density localization in the complexes in the solution alters the screening effect experienced by each NMR nucleus resulting in a different NMR response [46]. NMR peaks predicted from DFT calculations can be used to complement the experimental results and distinguishing different species present in the solution. However, the NMR predicted shifts from DFT strongly depend on the reference compound peak and all the possible structures of ionic species, in principle, have to be accounted for. An in-depth understanding of the solvation structure and different species present in the solution and how these species affect the macroscopic performance of electrolyte solutions requires coupling simulations and experiments at different spatial and temporal scales.
